# Rescuing Mitochondrial Dysfunction in Macrophages Prevents Osteonecrosis of the Jaw in Anti‐Resorptive Therapy

**DOI:** 10.1002/advs.202517586

**Published:** 2025-12-07

**Authors:** Hang Zhang, Xin Shen, Haiyang Liu, Xinxi Yuan, Mumin Cao, Xuepeng Lv, Ziji Ling, Songsong Guo, Rongyao Xu, Xiang Li, Hongbing Jiang

**Affiliations:** ^1^ State Key Laboratory Cultivation Base of Research Prevention and Treatment for Oral Diseases Nanjing Medical University Nanjing 210029 China; ^2^ Department of Oral and Maxillofacial Surgery Affiliated Hospital of Stomatology Nanjing Medical University Nanjing 210029 China; ^3^ Jiangsu Province Engineering Research Center of Stomatological Translational Medicine Nanjing Medical University Nanjing 210029 China; ^4^ Department of Orthopaedics Orthopaedic Trauma Institute (OTI) Trauma Center Zhongda Hospital School of Medicine Southeast University Nanjing 210009 China; ^5^ State Key Lab of Digital Medical Engineering Jiangsu Laboratory for Biomaterials and Devices School of Biological Science and Medical Engineering Southeast University Nanjing 211189 China

**Keywords:** anti‐resorptive therapy, autophagy, bisphosphonate‐related osteonecrosis of the jaw, macrophage polarization, mitochondrial dysfunction, PI3K‐AKT‐mTOR pathway, zoledronic acid

## Abstract

Mitochondria‐driven macrophage dysregulation contributes significantly to inflammatory disease progression; however, the mechanism underlying bisphosphonate‐related osteonecrosis of the jaw (BRONJ) remains unclear. This study demonstrates that zoledronic acid (ZA) disrupts mitochondrial bioenergetic function in macrophages, leading to elevated mitochondrial membrane potential, excessive mitochondrial reactive oxygen species (mtROS), and increased HIF‐1α expression, which together promote a pro‐inflammatory transition in macrophages. ZA further inhibits autophagy by activating the TLR4‐MyD88/PI3K‐AKT‐mTOR pathway, preventing the clearance of dysfunctional mitochondria and sustaining superoxide production. Genetic loss of *Atg5* in innate immune cells disrupts autophagosome maturation and markedly worsens ZA‐induced BRONJ development. To restore mitochondrial degradation and biofunction, ZA‐loaded nanoparticles incorporating the mTOR inhibitor rapamycin (ZDPR) are developed. ZDPR effectively prevents BRONJ and exerts therapeutic benefits in osteoporosis and osteolysis. These findings highlight bone‐targeted mitochondria rescue as a promising strategy to enhance antiresorptive therapy.

## Graphical abstract

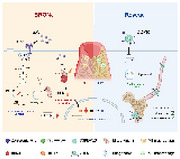

## 1 Introduction

Bisphosphonates (BPs) are widely used to treat bone resorptive diseases such as osteoporosis, Paget's disease, cancer bone metastases, and multiple myeloma.^[^ ^1^, ^2^, ^3^, ^4^ ^]^ They inhibit bone resorption by binding to mineralized bone surfaces and suppressing osteoclast activity through farnesyl diphosphate synthase (FDPS) inhibition in the mevalonate pathway.^[^ ^5^ ^]^ However, prolonged use of potent BPs, such as zoledronic acid (ZA), can lead to a severe complication known as bisphosphonate‐related osteonecrosis of the jaw (BRONJ), characterized by gingival dehiscence, necrotic bone exposure, and infection.^[^ ^6^ ^]^ Therefore, investigating its underlying mechanisms is essential to inform the development of safer therapeutic alternatives.

Bone modeling governs skeletal development and homeostasis, by balancing osteoclast‐mediated bone resorption, osteoblast‐mediated bone formation, and blood supply to ensure skeletal integrity and osteogenesis.^[^ ^7^ ^]^ In BRONJ, osteoclasts are the first to internalize bone‐bound ZA, resulting in suppressed bone turnover and compromised jawbone vascularization, which promotes tissue necrosis. Recent findings reveal multiple osteal macrophages that form specific niches in bone marrow, where they interact with osteoclasts and other cells, serving as important regulators of bone remodeling.^[^ ^8^, ^9^ ^]^ Our previous study showed that ZA‐treated macrophages suppress osteoclast differentiation and type H vessel formation during tooth extraction socket healing.^[^ ^10^ ^]^ These observations emphasize the pivotal role of macrophages in coupling osteogenesis and angiogenesis in BRONJ pathogenesis. However, the mechanism by which ZA disrupts microenvironmental homeostasis remains unclear.

As key regulators of inflammation, macrophages maintain tissue homeostasis through dynamic plasticity across a spectrum of phenotypes in response to various environmental cues.^[^ ^11^ ^]^ Upon activation, they secrete cytokines such as IL‐1β, IL‐6, and TNF‐α, contributing to the initiation and progression of bone inflammation.^[^ ^12^ ^]^ Recent studies indicate that macrophage pro‐inflammatory polarization, excessive IL‐1β secretion, and sustained tissue inflammation drive BRONJ development.^[^ ^13^, ^14^, ^15^, ^16^, ^17^ ^]^ Macrophage depletion worsens BRONJ‐like lesions, accompanied by severe osteoclast inhibition and pro‐inflammatory shifting.^[^ ^18^ ^]^ Our previous work demonstrated TLR4‐mediated macrophage M1‐like polarization in BRONJ using a TLR4‐knockout mouse model;^[^ ^19^ ^]^ however, the mechanism underlying ZA‐induced macrophage dysregulation remains unclear.

Because mitochondrial metabolism regulates macrophage polarization and immune function, the interaction between mitochondrial bioenergetics and mitochondrial reactive oxygen species (mtROS) signaling is critical for macrophage phenotype determination.^[^ ^20^ ^]^ As dynamic organelles generating ATP via oxidative phosphorylation, mitochondria face constant endogenous and exogenous stressors, requiring quality control systems to maintain functional integrity.^[^ ^21^ ^]^ Autophagy enables lysosomal degradation of intracellular materials, acting as an evolutionarily conserved mechanism that recycles cellular components and removes excessive or damaged organelles.^[^ ^22^ ^]^ Mitophagy, a selective form of autophagy that targets damaged mitochondria for autophagosomal capture, depends on efficient mitochondria priming, adaptor protein recognition, phagophore expansion, and the core autophagy machinery for autophagosome formation.^[^ ^23^, ^24^ ^]^ Notably, defective mitophagy characterizes activated macrophages,^[^ ^25^ ^]^ and autophagic flux is impaired in the gingival epithelium during BRONJ.^[^ ^26^ ^]^ Therefore, further investigation is required to clarify the roles of mitochondrial dysfunction and autophagy/mitophagy in macrophage polarization and BRONJ pathogenesis.

In this study, we examined mitochondrial dysfunction in macrophages during ZA‐induced BRONJ and explored a synergistic strategy to prevent this complication in bone resorptive disease treatment. Our findings enhance understanding of BRONJ pathogenesis and identify bone‐targeted mitochondrial rescue as a promising approach for antiresorptive therapy.

## 2 Results

### 2.1 ZA Induces Sustained Pro‐Inflammatory Polarization of Macrophages to Drive BRONJ Pathogenesis

**Figure 1:**
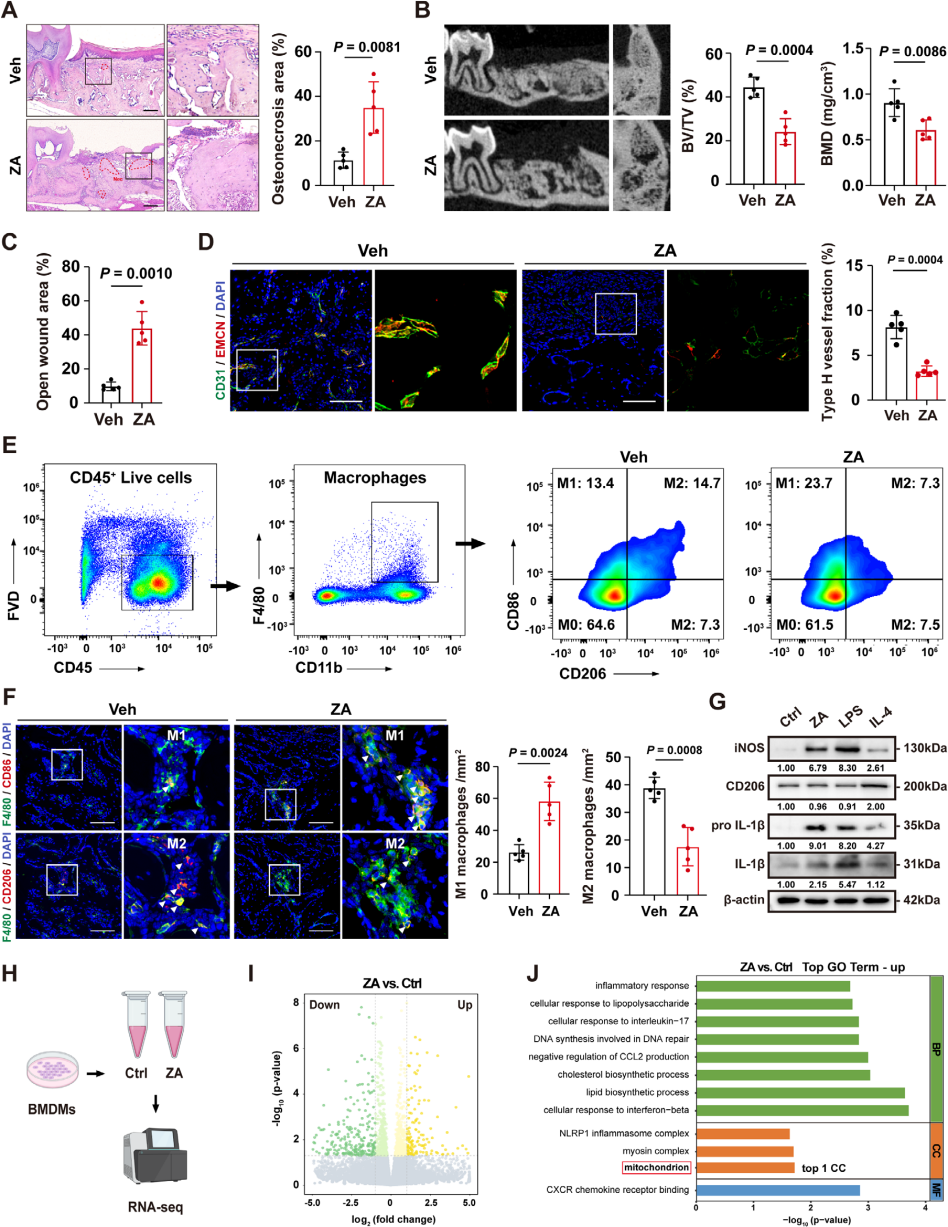
ZA promotes macrophage pro‐inflammatory polarization and induces BRONJ. A) Representative H&E‐stained images of tooth extraction site (TES). Red dashed lines indicate osteonecrosis area (Nec). Scale bars: 200 µm. B) Micro‐CT analysis of new bone formation in TES. C) The panel shows the quantitative data of open wound area (*n* = 5). D) Immunofluorescence (IF) imaging of CD31^hi^ EMCN^hi^ type‐H vessels in tooth extraction socket (TES). Scale bars: 100 µm. E) Gating strategy and flow cytometry assessment of jawbone macrophage stained with CD45, CD11b, F4/80, CD86, and CD206 antibodies. F) IF assay of F4/80^+^CD86^+^ or F4/80^+^CD206^+^ macrophages in TES. White arrows indicate representative cells. Scale bars: 100 µm. G) Western blot revealing the total protein expression of iNOS, CD206, pro IL‐1β, and IL‐1β in BMDMs treated with ZA, LPS, or IL‐4 for 24 h. H) Schematic depicting the bulk RNA sequencing process for Ctrl or ZA‐treated BMDMs. I) Volcano plot of gene expression levels determined by bulk RNA sequencing. Deep green and yellow plots represent genes that were significantly differentially expressed. J) Top pathways identified by GO enrichment analysis from differentially expressed genes of BMDMs after ZA treatment. Results are representative of at least three independent experiments performed. Densitometric quantifications are indicated beneath the representative blots. Panels show the quantitative data of osteonecrosis area (A, *n* = 5), trabecular bone volume/tissue volume (BV/TV), and bone mineral density (BMD) in TES (B, *n* = 5), type‐H vessel fraction (D, *n* = 5), and M1‐like and M2‐like macrophage numbers (F, *n* = 5). Statistical significance was examined by two‐tailed Student's *t*‐tests or a two‐sided unpaired Welch's *t*‐tests (A–D, F). Results are presented as the mean ± SD.

Following our established protocol,^[^ ^10^, ^19^ ^]^ the BRONJ‐like condition was reproduced in a murine model (Figure S1A, Supporting Information). Mice administered the vehicle solution exhibited normal healing of the tooth extraction sockets (TES), with gingival closure occurring within one week. In contrast, ZA‐treated mice showed persistent TES openings for up to three weeks, necrotic bone formation (**Figure** **1**A–C; Figure S1B, Supporting Information), and reduced bone mineral density (BMD) and bone volume fraction (BV/TV) (Figure 1B). Immunolabeling and tartrate‐resistant acid phosphatase (TRAP) staining revealed marked reductions in CD31^hi^/EMCN^hi^ type H vessels and osteoclasts in ZA‐treated mice compared to PBS‐treated controls (Figure 1D; Figure S1C, Supporting Information). Quantification of the main immune cell types in TES showed a significant increase in macrophages (Figure S1D, Supporting Information). The ZA group displayed a notable rise in M1‐like polarized macrophages (F4/80^+^CD86^+^) and a concurrent decline in M2‐like polarization (F4/80^+^CD206^+^) (Figure 1E,F; Figure S1E, Supporting Information). Normally, lipopolysaccharides (LPS) induce a pro‐inflammatory phenotype, while interleukin‐4 (IL‐4) promotes alternative polarization.^[^ ^27^ ^]^ In vitro, ZA‐treated bone marrow‐derived macrophages (BMDMs) exhibited classical activation features compared to LPS or IL‐4 stimulation (Figure 1G) and a higher M1/M2 ratio (Figure S1F, Supporting Information). These results indicate that ZA promotes pro‐inflammatory macrophage polarization in vivo and in vitro.

To further explore ZA's effects on macrophages, RNA sequencing was performed on BMDMs 12 h after ZA stimulation (Figure 1H,I). Gene Ontology (GO) analysis revealed upregulation of genes related to pro‐inflammatory responses and cytokine production (Figure 1J). Notably, mitochondria were the most enriched cellular component (Figure 1J; Figure S1G, Supporting Information), suggesting mitochondrial reorganization during ZA‐induced pro‐inflammatory polarization. Together, these findings demonstrate that ZA‐induced macrophage polarization involves enhanced expression of inflammation‐ and mitochondria‐related genes.

### 2.2 ZA Enhances Macrophage ΔΨm and mtROS Production, thereby Activating the Pro‐Inflammatory Transcription Factor HIF‐1α

**Figure 2:**
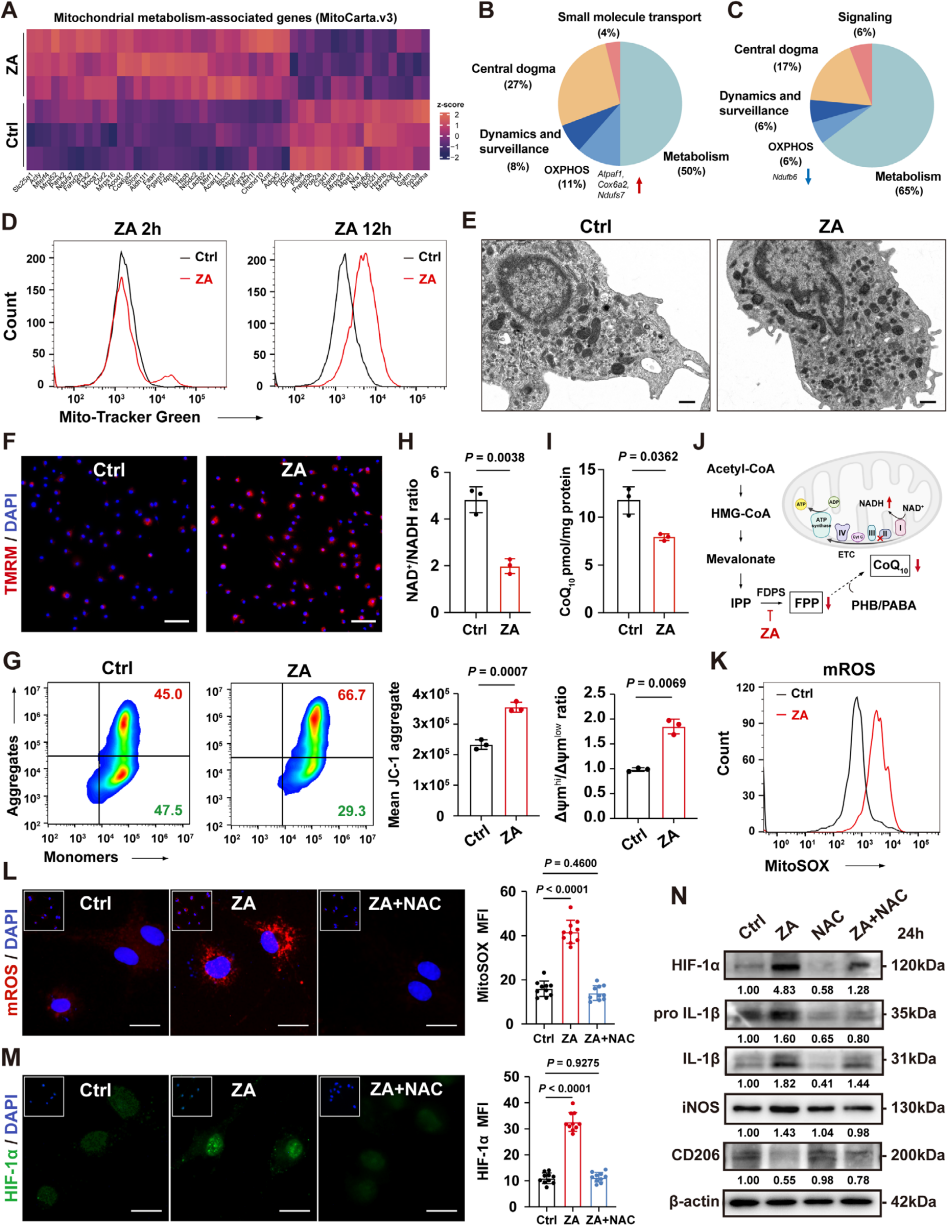
ZA enhances macrophage ΔΨm and mtROS production, thus activating pro‐inflammatory signal HIF‐1α. A) Heat maps showing the top differentially expressed mitochondria‐associated genes in BMDMs in Ctrl or ZA groups. Up B) and down C) regulated genes in the ZA group categorized by their function. D) Flow cytometry assessment of mitochondrial density in BMDMs at the indicated time points. E) Representative TEM images of mitochondria in BMDMs. Scale bars: 500 nm. F) TMRM assay kit reflecting mitochondrial membrane potential in BMDMs treated with ZA. Scale bars: 50 µm. G) Flow cytometry of JC‐1 staining detecting the intracellular JC‐1 aggregates and monomers in BMDMs. H) NAD^+^/NADH assay kit measuring the intracellular NAD^+^/NADH ratio in BMDMs (*n = *3). I) Coenzyme Q10 assay kit measuring the intracellular CoQ_10_ level in BMDMs (*n = *3). J) Schematic of the inhibition effect of ZA on ETC and CoQ_10_ biosynthesis by targeting the mevalonate pathway. K) Flow cytometry assessment of mitochondrial ROS in BMDMs. L,M) mtROS and HIF‐1α expression in BMDMs treated with ZA only, or with ZA+NAC for 24 h. Scale bars: 20 µm. N) Western blot revealing HIF‐1α, pro IL‐1β, IL‐1β, iNOS, and CD206 protein expression in BMDMs treated with ZA only, NAC only, or ZA+NAC for 24 h. Results are representative of at least three independent experiments performed. Densitometric quantifications are indicated beneath the representative blots. Panels show the quantitative data of mean expression level of JC‐1 aggregates (G, *n* = 3) and JC‐1 aggregates/monomers ratio (G, *n* = 3), mean MitoSOX Red (L, *n* = 10), and mean HIF‐1α expression intensity (M, *n* = 10). Statistical significance was examined by a two‐tailed Student's *t*‐tests or two‐sided unpaired Welch's *t*‐tests (G–I), and two‐sided one‐way ANOVA or Welch ANOVA (L,M). Results are presented as the mean ± SD.

Given the close relationship between macrophage polarization and mitochondrial bioenergetic function, we hypothesized that mitochondrial metabolic reprogramming is a key mechanism underlying ZA‐driven pro‐inflammatory polarization. To test this, we analyzed the effects of ZA on mitochondrial protein‐encoding genes listed in MitoCarta.^[^ ^28^ ^]^ Transcriptomic analysis identified 32 upregulated and 17 downregulated genes (**Figure** **2**A), many of which were linked to metabolism and OXPHOS (Figure 2B,C). Mitochondrial density, assessed using the membrane potential (ΔΨm) insensitive dye MitoTracker Green and flow cytometry, was significantly increased at 12 h (Figure 2D; Figure S2A, Supporting Information). Transmission electron microscopy (TEM) confirmed greater mitochondrial density and a shift toward a more spherical morphology in ZA‐treated cells (Figure 2E; Figure S2B, Supporting Information).

To assess mitochondrial respiration, we used a Seahorse XFe96 analyzer to measure the oxygen consumption rate (OCR) of BMDMs. A 24‐h ZA pretreatment markedly reduced resting oxygen consumption, maintaining low levels for the next 5 h (Figure S2E, Supporting Information). The NAD^+^/NADH ratio, an indicator of electron transport chain (ETC) activity,^[^ ^29^ ^]^ significantly decreased (Figure 2H). This suggests impaired NADH oxidation and diminished ATP synthesis. Paradoxically, despite reduced respiration, TMRM staining showed elevated ΔΨm in ZA‐treated macrophages (Figure 2F; Figure S2C, Supporting Information). Flow cytometry of JC‐1 staining further confirmed an increased proportion of high‐ΔΨm mitochondria after ZA exposure (Figure 2G). Normally, ΔΨm maintenance depends on an active ETC and efficient oxidative phosphorylation.^[^ ^30^ ^]^ We therefore hypothesized that ZA disrupts ETC complex activity by impairing coenzyme Q10 biosynthesis. Consistent with this, ZA treatment decreased the coenzyme Q10 pool in BMDMs (Figure 2I,J). These results correspond with prior evidence that activated macrophages elevate ΔΨm and enhance mtROS production at complex I to regulate immune activation and cytokine signaling.^[^ ^31^, ^32^, ^33^ ^]^ MitoSOX Red staining revealed a marked increase in mtROS levels in ZA‐treated BMDMs (Figure 2K,L; Figure S2D, Supporting Information).

Mitochondrial dysfunction and excessive mitochondrial superoxide production stabilize HIF‐1α, a key pro‐inflammatory transcription factor that regulates cytokine expression and inflammatory signaling.^[^ ^34^ ^]^ Consistent with this mechanism, our findings showed that ZA treatment increased HIF‐1α expression, which was reversed by the ROS scavenger N‐acetyl‐L‐cysteine (NAC). Concurrently, both pro IL‐1β and IL‐1β expression levels were elevated (Figure 2M,N). These results demonstrate that ZA enhances mitochondrial dysfunction and mtROS production, thereby promoting HIF‐1α activation and contributing to a pro‐inflammatory macrophage phenotype.

### 2.3 ZA Blocks ΔΨm‐Dependent PINK1/Parkin Recruitment and Disrupts Downstream Autophagic Flux

**Figure 3:**
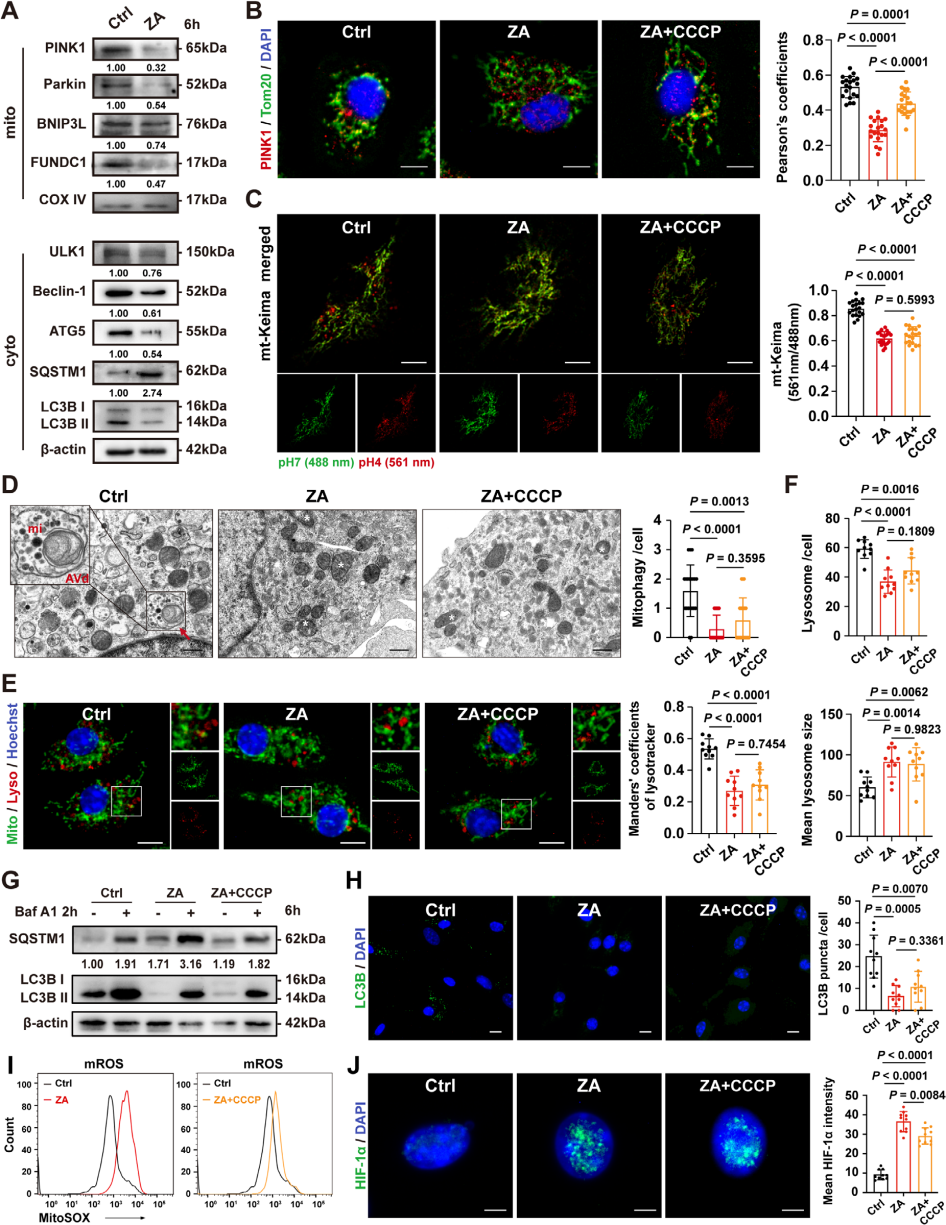
Inhibition of autophagic flux instead of PINK1/Parkin recruitment is a main source for mtROS production A) Western blot revealing the mitochondrial protein expression of PINK1, Parkin, BNIP3L, FUNDC1, and total protein expression of ULK1, Beclin‐1, ATG5, SQSTM1, and LC3B in BMDMs. B) Immunofluorescence (IF) staining showing the co‐localization of PINK1/Tom20 in BMDMs treated with ZA only, or ZA+CCCP for 6 h. Scale bars: 10 µm. C) Fluorescence imaging of mitophagy events in BMDMs expressing mt‐Keima, and treated with ZA only, or ZA + CCCP for 6 h. Scale bars: 10 µm. D) Representative TEM images of BMDMs treated with ZA only, or ZA + CCCP for 6 h. Red arrow indicates the typical formation of mitophagic vacuoles. White asterisks indicate damaged mitochondria. Zoomed boxed area presents a magnified structure of a degradative autophagic vacuole (AVd) containing a mitochondrion (mi). Scale bars: 500 nm. E) Representative images of BMDMs stained with MitoTracker Green (mitochondria) and LysoTracker Red (lysosomes). Scale bars: 10 µm. F) Panels show the quantitative data of lysosome number per cell (*n* = 10), and average size (*n* = 10). G) Western blot revealing total protein expression of SQSTM1, and LC3B in BMDMs treated with indicated interventions for 6 h. H) IF staining showing LC3B expression in BMDMs treated with ZA only, or ZA + CCCP. Scale bars: 10 µm. I) Flow cytometry assessment of mitochondrial ROS in BMDMs treated with ZA only, or ZA + CCCP. J) HIF‐1α expression in BMDMs treated with ZA only, or ZA + CCCP for 24 h. Scale bars: 5 µm. Results are representative of at least three independent experiments performed. Densitometric quantifications are indicated beneath the representative blots. Panels show the quantitative data of the Pearson's coefficients of PINK1/Tom20 (B, *n* = 20), the ratio of signal excited at 561 and 488 nm (C, *n* = 20), mitophagy events per cell (D, *n* = 20), Manders’ coefficients of lysotracker (E, *n* = 10), LC3B puncta per cell (H, *n = *10), and mean expression level of HIF‐1α (J, *n* = 10). Statistical significance was examined by a two‐sided one‐way ANOVA or Welch ANOVA (B–F,H,J). Results are presented as the mean ± SD (^***^ *p* <0.001; ns *p* >0.05).

The observed increase in mitochondrial density and mtROS generation following ZA exposure likely results from defective mitochondrial clearance rather than enhanced biogenesis, which generally occurs more slowly.^[^ ^35^ ^]^ Because mitochondrial depolarization is essential for PINK1/Parkin‐dependent mitophagy initiation, we hypothesized that ZA‐induced elevation of ΔΨm prevents depolarization, thereby perpetuating a self‐sustaining loop of ΔΨm/mtROS/HIF‐1α activation and mitophagy inhibition. To test this, mitochondrial fractionation assays were performed to evaluate the recruitment of mitochondrial damage sensors. ZA treatment significantly decreased the mitochondrial localization of PINK1, Parkin, BNIP3L, and FUNDC1. The total cellular levels of ULK1 and Beclin‐1—key regulators of autophagosome formation—were also reduced as early as 6 h post‐treatment. Additionally, the accumulation of the selective autophagy adaptor SQSTM1 (sequestosome 1), reduced ATG5 expression, and a lower LC3B II/I ratio were observed (**Figure** **3**A). Immunofluorescence (IF) staining confirmed a marked reduction in PINK1 co‐localization with the outer mitochondrial membrane marker Tom20 after 6 h of ZA exposure (Figure 3B).

Interestingly, although the mitochondrial uncoupler carbonyl cyanide m‐chlorophenyl hydrazone (CCCP) efficiently reduced ΔΨm and promoted PINK1 recruitment (Figure 3B; Figure S3A, Supporting Information), mitophagy remained inhibited in BMDMs expressing the mt‐Keima reporter after treatment with either ZA or ZA + CCCP (Figure 3C). The reduced mt‐Keima signal at 561 nm indicated impaired mitochondria delivery to acidic lysosomes. TEM further revealed a marked decline in mitophagic vacuoles (double‐membrane mitophagosomes containing mitochondria‐like structures or remnants of mitochondrial cristae). Additionally, there was an accumulation of damaged mitochondria characterized by swelling, matrix vacuolization, and disrupted cristae in both ZA and ZA + CCCP groups (Figure 3D). MitoTracker Green and LysoTracker Red staining revealed reduced mitochondria–lysosome co‐localization and alterations in both the number and size of lysosomes (Figure 3E,F). These findings suggest that ZA‐induced mitophagy suppression is not primarily mediated by high ΔΨm or impaired PINK1/Parkin recruitment. To further investigate downstream autophagic flux, the lysosomal inhibitor bafilomycin A1 (Baf A1) was applied for 2 h. Compared with Baf A1 alone, the ZA + Baf A1 group exhibited decreased LC3B‐II levels, while CCCP treatment failed to increase LC3B‐II expression (Figure 3G). IF staining further demonstrated a marked reduction in intracellular LC3B puncta in BMDMs (Figure 3H), indicating inhibition of autophagosome biogenesis. Furthermore, CCCP did not reverse the elevated mtROS levels, increased HIF‐1α expression, or the pro‐inflammatory activation of BMDMs (Figure 3I,J; Figure S3B,C, Supporting Information). Collectively, these results indicate that ZA not only disrupts ΔΨm‐dependent mitophagy initiation, but more importantly, impairs downstream autophagic flux, thereby preventing lysosomal degradation of damaged mitochondria.

### 2.4 ZA Impairs Mitochondrial Clearance by Inhibiting Autophagy Machinery via the TLR4‐MyD88/PI3K‐AKT‐mTOR Axis

**Figure 4:**
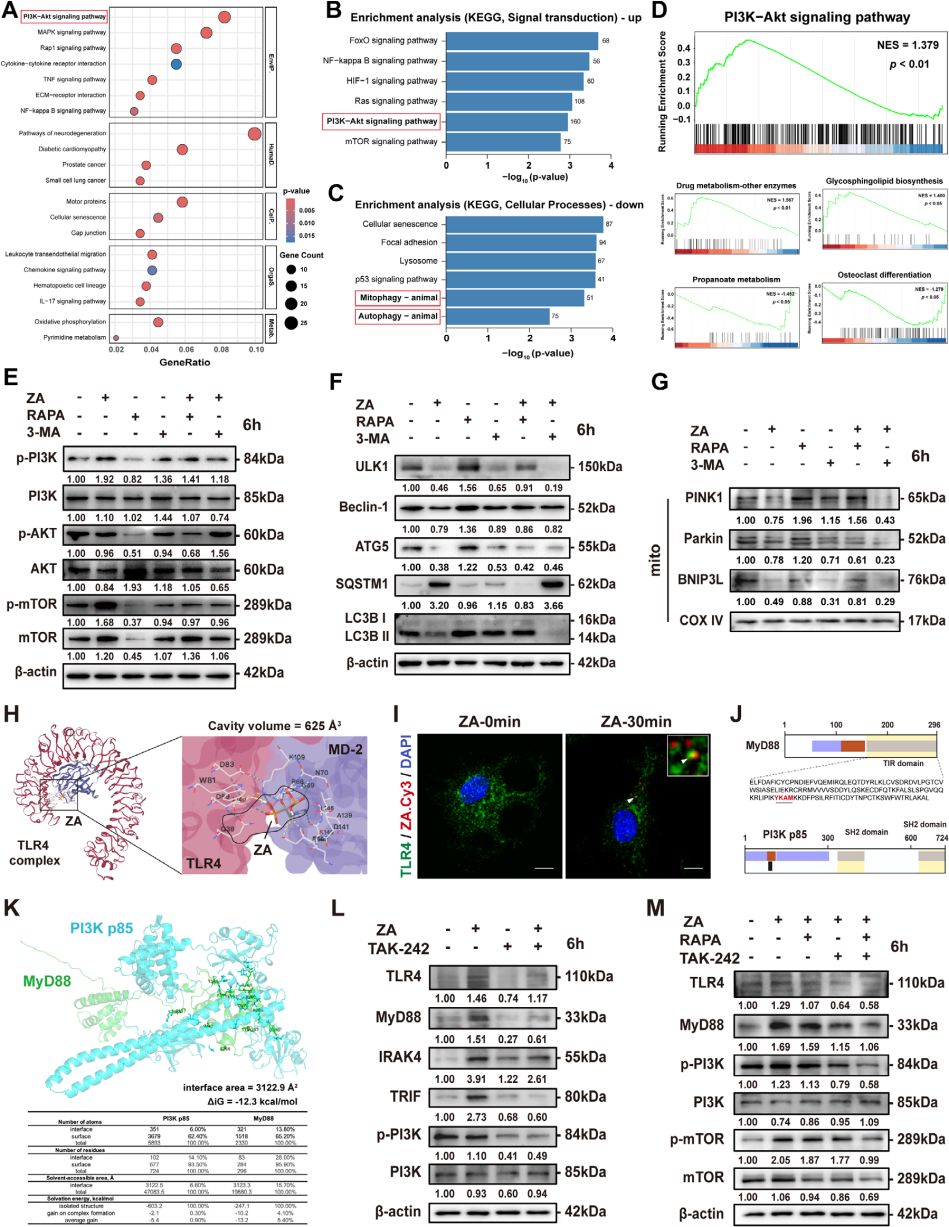
ZA inhibits autophagy machinery via TLR4‐MyD88/PI3K‐AKT‐mTOR and triggers pro‐inflammatory activation of macrophages. Top pathways identified by KEGG enrichment analysis from the total differentially expressed genes A), upregulated genes B), and downregulated genes C) of BMDMs after ZA treatment. D) Top pathways identified by gene set enrichment analysis of BMDMs after ZA treatment. Total protein expressions of PI3K‐AKT‐mTOR pathway E), autophagic flux markers F), and mitochondrial adaptor proteins G) in BMDMs with indicated treatments for 6 or 24 h. H) Molecular docking simulations of ZA and the murine TLR4 complex. I) Immunofluorescence (IF) staining showing the co‐localization of ZA/TLR4 in BMDMs treated with ZA‐Cy3 for 30 min. Scale bars: 10 µm. J) Amino acid residue sequences of MyD88 and PI3K p85 subunit. K) Determination of the MyD88‐PI3K protein interaction surface. Table below shows the detailed docking information. L,M) Total protein expression of the TLR4 pathway and PI3K‐AKT‐mTOR pathway in BMDMs with indicated treatments for 6 h. Results are representative of at least three independent experiments performed. Densitometric quantifications are indicated beneath the representative blots.

To elucidate the mechanism by which ZA disrupts mitochondrial sequestration and degradation, RNA‐seq analysis was performed. The results revealed significant enrichment of the PI3K‐AKT and mTOR signaling pathways among the top‐ranked signal transduction categories (**Figure** **4**A–C). Gene set enrichment analysis (GSEA) further confirmed a strong positive correlation between ZA treatment and the PI3K‐AKT/mTOR pathway gene sets (Figure 4D). The mTOR pathway is a key negative regulator of autophagy and lysosomal biogenesis^[^ ^36^ ^]^ and inhibits the upstream autophagy‐initiating complex and subsequent recruitment of autophagy‐related proteins, thereby hindering selective mitochondria clearance.^[^ ^37^ ^]^ Western blot analysis validated these findings by demonstrating increased phosphorylation of PI3K and its downstream target AKT and mTOR following ZA treatment (Figure 4E). In the presence of the autophagy inhibitor 3‐Methyladenine(3‐MA) or the activator rapamycin (RAPA), an mTOR inhibitor, a synergistic inhibition of autophagic flux was observed in the ZA and 3‐MA groups, whereas RAPA suppressed mTOR phosphorylation and promoted the recruitment of the transmembrane receptor BNIP3L to the mitochondria (Figure 4E–G). Correspondingly, the protein expression levels of ULK1, ATG5, LC3B II/I ratio, and SQSTM1 were altered (Figure 4F). mt‐Keima and TEM imaging revealed that RAPA was more effective in promoting lysosomal degradation of mitochondria (Figure S4B,C, Supporting Information) and alleviating hyperactivation of the mtROS/HIF‐1α axis (Figure S4D–F, Supporting Information). Notably, RAPA also suppressed ZA‐induced inflammatory cytokine production (Figure S4A, Supporting Information).

Our previous research demonstrated that ZA activates the TLR4 pathway to promote a pro‐inflammatory phenotype.^[^ ^19^ ^]^ TLR4 recognizes PAMPs and recruits the adaptor protein MyD88 to initiate downstream signaling cascades involving multiple pathways, including PI3K. Studies have shown that the SH2 domains in the p85 regulatory subunit of PI3K can directly bind to the “YXXM” motif within the Toll/IL‐1 receptor domain of MyD88.^[^ ^38^, ^39^, ^40^ ^]^ Thus, ZA‐induced PI3K phosphorylation may be associated with TLR4‐MyD88 activation. Molecular docking simulations using CB‐Dock2 revealed stable binding of ZA to the murine TLR4/MD‐2 complex (Figure 4H). Consistent with this finding, IF staining showed close co‐localization of ZA‐Cy3 and TLR4 receptor 30 min after treatment with the fluorescent ZA analog, suggesting active TLR4 involvement in ZA recognition and internalization (Figure 4I). Amino acid sequence analysis of MyD88 and p85 subunit, identified docking of the YXXM motif at residues Y^257^–M^260^ within the SH2 domains of p85 (Figure 4J,K). Western blot analysis confirmed that ZA activated TLR4 and upregulated the adaptor proteins MyD88, IRAK4, and TRIF, whereas the selective TLR4 inhibitor TAK‐242 attenuated this response and decreased PI3K phosphorylation (Figure 4L). RAPA did not significantly affect TLR4 signaling but directly inhibited mTOR activity (Figure 4M). Collectively, these results demonstrate that ZA inhibits the core autophagy machinery through the TLR4‐MyD88/PI3K‐AKT‐mTOR pathway, while RAPA exerts a rescuing effect by restoring autophagic function.

### 2.5 Deficiency of Autophagosome Biogenesis in *Lyz2cre; Atg5^f/f^* Mouse Exacerbates BRONJ‐Like Lesions

**Figure 5:**
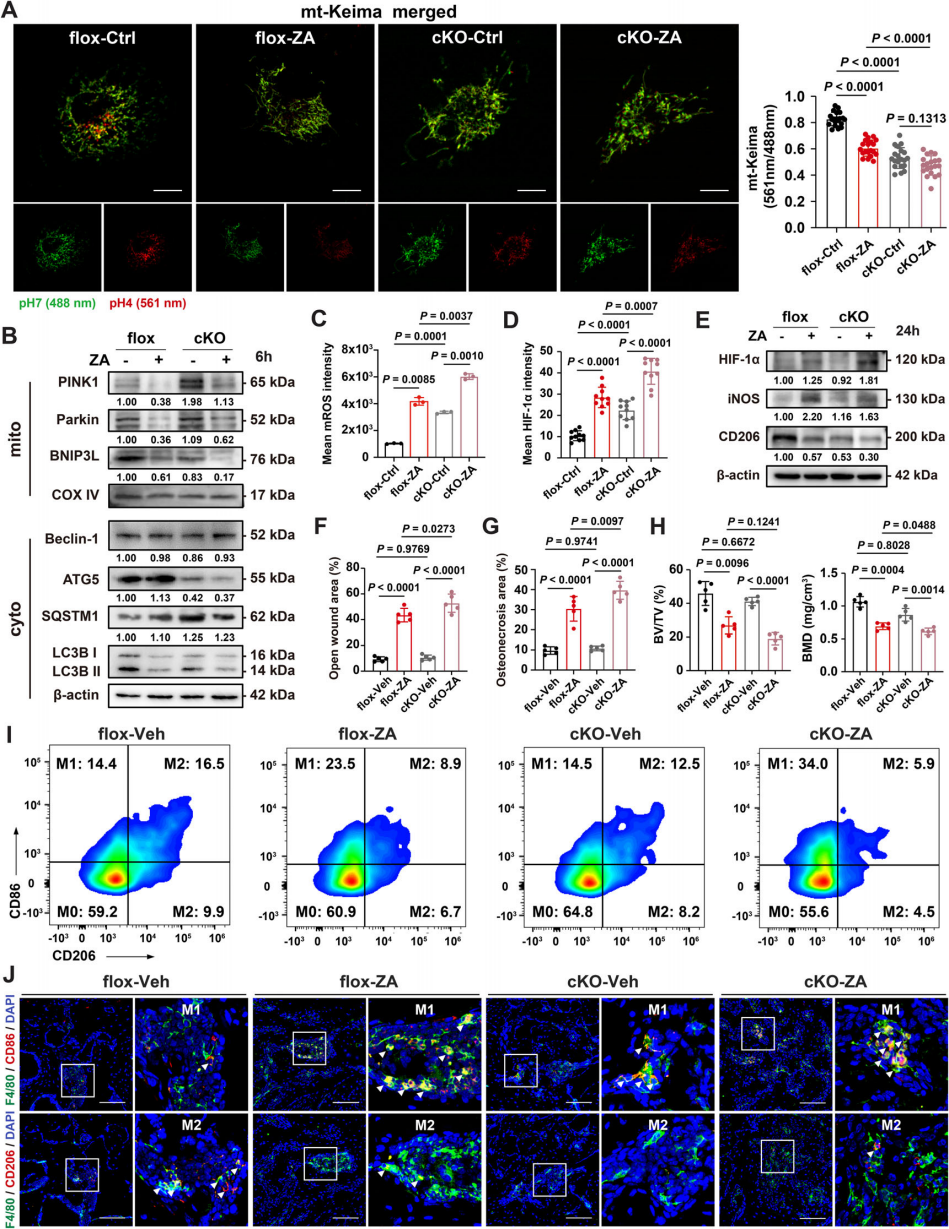
Deficiency of autophagosome maturation in *Lyz2cre; Atg5^f/f^* mouse exacerbates BRONJ‐like lesion. A) Fluorescence imaging of mitophagy events in flox or cKO BMDMs expressing mt‐Keima with or without ZA treatment for 6 h. The right panel shows quantitative measurements for the ratio of signal excited at 561 and 488 nm (*n* = 20). Scale bars: 10 µm. B) Total protein expression of autophagic flux markers, and mitochondrial adaptor proteins in flox or cKO BMDMs with or without ZA treatment for 6 h. C,D) Panels show quantitative data of mean mtROS (*n* = 3) and mean HIF‐1α expression (*n* = 3) in flox or cKO BMDMs with or without ZA treatment. Scale bars: 5 µm. E) Total protein expression of HIF‐1α, iNOS, and CD206 in flox or cKO BMDMs with or without ZA treatment for 24 h. F–H) Panels show the quantitative data of open wound area (*n* = 5), osteonecrosis area (*n* = 5), trabecular bone volume/tissue volume (BV/TV) (*n* = 5), and bone mineral density (BMD) (*n* = 5). I) Flow cytometry assessment of jawbone macrophage stained with CD45, CD11b, F4/80, CD86, and CD206 antibodies. J) Immunofluorescence (IF) assay of F4/80^+^CD86^+^ or F4/80^+^CD206^+^ macrophages in TES. White arrows indicate representative cells. Scale bars: 100 µm. Results are representative of at least three independent experiments performed. Densitometric quantifications are indicated beneath the representative blots. Statistical significance was examined by a two‐sided one‐way ANOVA or Welch ANOVA (A,C,D,F–H). Results are presented as the mean ± SD.

Downstream autophagosome formation, rather than conventional mitophagy initiation, represents a key target for further investigation, given that ZA inhibits mTOR and disrupts the core autophagy machinery. We generated macrophage‐specific conditional *Atg5* knockout mice by crossbreeding *Atg5^f/f^* and *Lyz2cre* strains (Figure S5A, Supporting Information), and BMDMs from *Atg5^f/f^* (flox) and *Lyz2cre; Atg5^f/f^* (cKO) mice were isolated for in vitro experiments. *Atg5* deficiency in BMDMs markedly impaired lysosomal mitochondrial degradation, evidenced by a reduced mt‐Keima signal at 561 nm (**Figure** **5**A). ZA‐treated *Atg5*‐deficient BMDMs exhibited severely impaired autophagy, the highest HIF‐1α expression (Figure 5B–E; Figure S5B, Supporting Information), and elevated iNOS protein levels (Figure 5E). We next induced BRONJ‐like lesions in flox and cKO mice (Figure S5C, Supporting Information). Although autophagy deficiency in macrophages alone did not induce BRONJ lesions, the cKO + ZA group displayed open extraction sockets and larger necrotic bone areas than the flox + ZA group (Figure 5F–H; Figure S5D–F, Supporting Information). Impaired autophagy hindered macrophage polarization toward an anti‐inflammatory phenotype following tooth extraction and exhibited a slight reduction in the abundance of CD31^hi^/EMCN^hi^ type H vessels, while the cKO + ZA group demonstrated significantly enhanced pro‐inflammatory responses and the worst vascularization (Figure 5I; Figure S5G, Supporting Information). Notably, a greater number of pro‐inflammatory macrophages were recruited to the TES of cKO mice compared with flox controls, and the cKO + ZA group exhibited the highest abundance of M1‐like macrophages (Figure 5J; Figure S5H, Supporting Information). Collectively, these results highlight the essential role of autophagy and selective mitochondria clearance in mitigating pro‐inflammatory tissue damage and angiogenesis inhibition during BRONJ pathogenesis.

### 2.6 Engineered ZDPR Nanoparticles Attenuate BRONJ through Rescuing Dysfunctional Mitochondria

**Figure 6:**
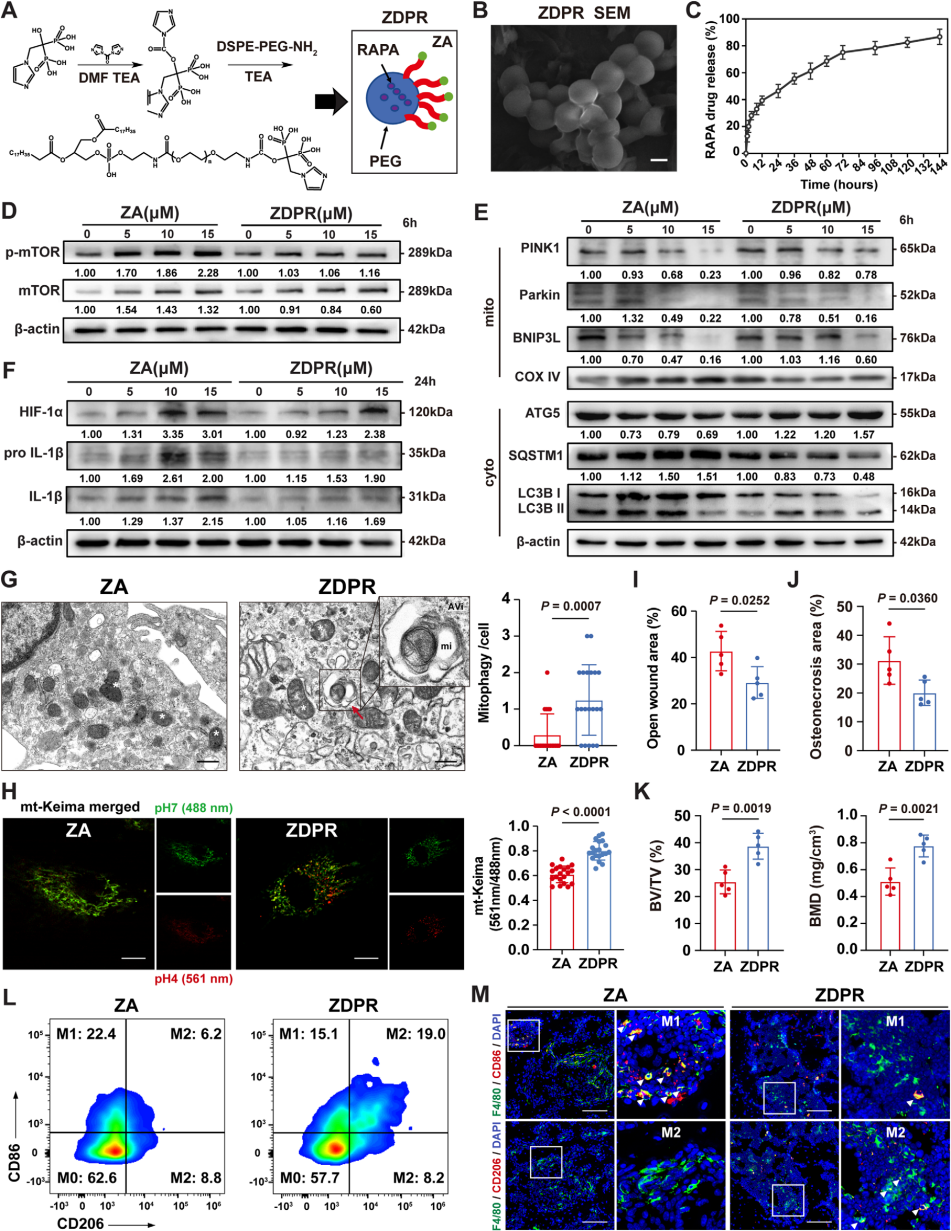
Synthesis of ZDPR and its preventing effects of BRONJ. A) Schematic illustration of the preparation of ZDPR. B) Scanning electron microscopy (SEM) image showing the surface morphology of ZDPR nanoparticles. Scale bars: 100 nm. C) Drug‐releasing profile of RAPA in ZDPR nanoparticles. D–F) Western blot revealing total protein expression of mTOR, p‐mTOR, autophagic flux markers, mitochondrial adaptor proteins, HIF‐1α, pro IL‐1β, and IL‐1β in BMDMs treated with 5, 10, 15 µm ZA or 5, 10, 15 µm ZDPR for 6 or 24 h. G) Representative TEM images of BMDMs treated with ZA or ZDPR for 6 h. Red arrow indicates the typical formation of mitophagic vacuoles. White asterisks indicate damaged mitochondria. Zoomed boxed area presents a magnified structure of an early autophagic vacuole (AVi) containing a mitochondrion (mi). Scale bars: 500 nm. H) Fluorescence imaging of mitophagy events in BMDMs expressing mt‐Keima with ZA or ZDPR treatment for 6 h. Scale bars: 10 µm. I–K) Panels show the quantitative data of open wound area (*n* = 5), osteonecrosis area (*n* = 5), trabecular bone volume/tissue volume (BV/TV) (*n* = 5), and bone mineral density (BMD) (*n* = 5). L) Flow cytometry assessment of jawbone macrophage stained with CD45, CD11b, F4/80, CD86, and CD206 antibodies. M) Immunofluorescence (IF) assay of F4/80^+^CD86^+^ or F4/80^+^CD206^+^ macrophages in TES. White arrows indicate representative cells. Scale bars: 100 µm. Results are representative of at least three independent experiments performed. Densitometric quantifications are indicated beneath the representative blots. Panels show the quantitative data of mitophagy events per cell (G, *n* = 20), and the ratio of signal excited at 561 and 488 nm (H, *n* = 20). Statistical significance was examined by a two‐tailed Student's *t*‐tests or two‐sided unpaired Welch's *t*‐tests (G–K). Results are presented as the mean ± SD.

ZA adsorption onto bone minerals is a principal cause of BRONJ.^[^ ^41^ ^]^ Given ZA's bone‐targeting property, we explored whether combining ZA with RAPA could alleviate its inhibitory effects on macrophage mitophagy within the jawbone. Polyethylene glycol conjugation (PEGylation) is a well‐established and safe method for drug delivery modification, and 1,2‐distearoyl‐sn‐glycero‐3‐phosphoethanolamine‐N‐[hydroxysuccinimidyl(polyethylene glycol)‐2000] (DSPE‐PEG_2000_‐NH_2_) is widely used owing to its amphiphilic characteristics.^[^ ^42^ ^]^ Therefore, RAPA‐DSPE_2000_‐PEG‐ZA (ZDPR) nanoparticles were engineered to facilitate bone‐targeted RAPA delivery (**Figure** **6**A). The conjugation of ZA with DSPE‐PEG_2000_‐NH_2_ was confirmed by nuclear magnetic resonance spectroscopy (Figure S6A, Supporting Information). DSPE‐PEG_2000_‐ZA conjugates were subsequently used to load RAPA, producing ZDPR nanoparticles with an entrapment efficiency of 79.84 ± 2.4% and a drug loading capacity of 7.41 ± 0.21% (Table S3, Supporting Information). Scanning electron microscopy verified the uniform morphology of the nanoparticles (Figure 6B), which exhibited an average diameter of 111.9 nm (Figure S6B, Supporting Information). The ZDPR nanoparticles displayed a prolonged drug release profile, characterized by an initial burst of ≈40% RAPA within the first 12 h, followed by a sustained release over the next 6 days (Figure 6C; Table S4, Supporting Information). The IC_50_ value of ZDPR was calculated to be 15.02 µm using the CCK‐8 assay (Figure S6C, Supporting Information). At a therapeutic concentration of 10 µm, ZDPR effectively inhibited the mevalonate pathway, comparable to traditional ZA, and suppressed osteoclastogenesis in vitro (Figure S6D,E, Supporting Information). Unlike ZA, ZDPR reduced mTOR phosphorylation (Figure 6D) and enhanced the mitophagic activity of BMDMs at equivalent concentrations (Figure 6E,G,H). Furthermore, ZDPR decreased HIF‐1α expression (Figure 6F) and promoted the polarization of BMDMs toward the anti‐inflammatory phenotype in vitro (Figure S6F,G, Supporting Information).

In a mouse BRONJ model, ZDPR markedly alleviated both the extent and severity of BRONJ‐like lesions (Figure 6I–K; Figure S6I–K, Supporting Information). Biodistribution analysis of rapamycin following intravenous administration of DSPE‐PEG‐RAPA or ZDPR nanoparticles revealed detectable accumulation in the jawbone in ZDPR groups compared with unconjugated DSPE‐PEG‐RAPA at 1, 4, and 24 h post‐injection (Figure S6H, Supporting Information). IF staining and flow cytometry confirmed a higher population of anti‐inflammatory macrophages within the TES in the ZDPR‐treated group (Figure 6L,M; Figure S6L, Supporting Information), and an increased type‐H vessel formation (Figure S6M, Supporting Information). Comprehensive histological examination of major organs showed no pathological abnormalities, indicating the absence of drug‐induced toxicity (data not shown). These findings collectively suggest that ZDPR effectively mitigates the occurrence and progression of BRONJ.

### 2.7 ZDPR Prevents the Development of Osteoporosis and Osteolytic Lesions

**Figure 7:**
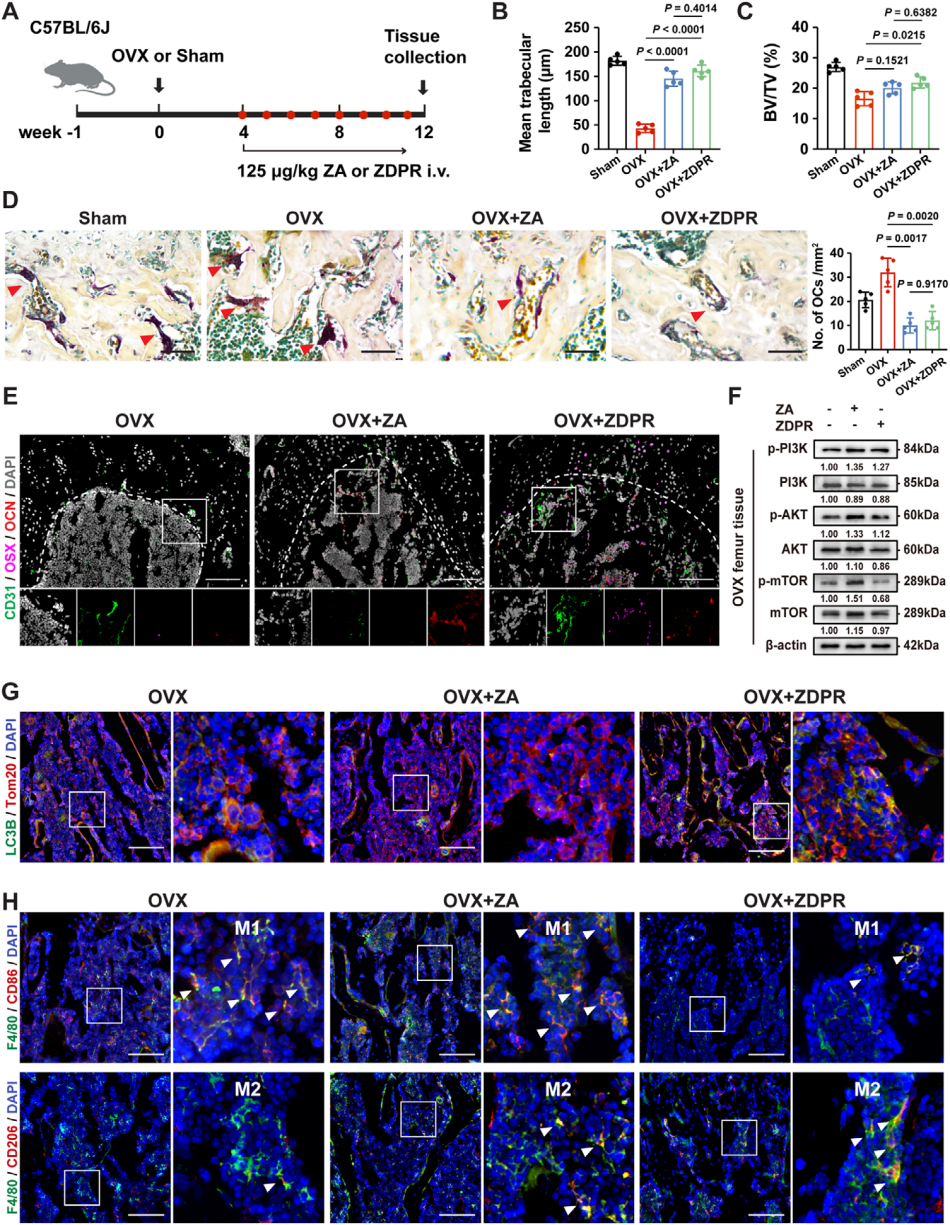
Efficacy of ZDPR in preventing osteoporosis. A) Experimental schedule for establishing an osteoporosis model in mice. B,C) Panels show the quantitative measurements of mean trabecular length of femur (*n* = 5), and trabecular bone volume/tissue volume (BV/TV) (*n* = 5). D) Representative TRAP‐stained images of femur 12 weeks after Sham, OVX operation, OVX and ZA injection, or OVX and ZDPR injection. The right panel shows the quantitative measurements of osteoclasts (*n* = 5). Scale bars: 50 µm. E) Immunofluorescence assay of femur 12 weeks after Sham, OVX operation, OVX and ZA injection, or OVX and ZDPR injection. White dashed lines indicate the epiphyseal line. Zoomed boxed areas present representative regions with positive staining of CD31 (green), OSX (magenta), and OCN (red). Scale bars: 100 µm. F) Tissue protein expression of the PI3K‐AKT‐mTOR pathway. G,H) Immunofluorescence (IF) staining showing the co‐localization of LC3B/Tom20 in femur, and F4/80^+^CD86^+^ or F4/80^+^CD206^+^ macrophages in femur. White arrows indicate representative cells. Scale bars: 100 µm. Results are representative of at least three independent experiments performed. Densitometric quantifications are indicated beneath the representative blots. Statistical significance was examined by a two‐sided one‐way ANOVA or Welch ANOVA (G–K). Results are presented as the mean ± SD.

To further assess ZDPR's therapeutic potential relative to ZA, we evaluated its efficacy in an ovariectomy (OVX)‐induced osteoporosis mouse model. After 8 weeks of treatment, the bone structure in the distal femur was analyzed (**Figure** **7**A). The OVX group demonstrated pronounced bone loss compared to the Sham group, indicated by significant reductions in trabecular bone volume and length (Figure 7B,C; Figure S7A,B, Supporting Information). Treatment with ZA or ZDPR preserved bone mass, with ZDPR showing a greater improvement in the BV/TV ratio and trabecular thickness compared to ZA (Figure S7B, Supporting Information). TRAP staining revealed that both ZA and ZDPR significantly reduced osteoclasts numbers, reflecting decreased bone resorption activity (Figure 7D). Interestingly, ZDPR treatment increased the perivascular expression of osteogenic markers, including osteocalcin (OCN) and osterix (OSX), indicating enhanced osteoblast lineage progression compared to the ZA group (Figure 7E). In addition, ZDPR reduced the protein expression of the PI3K‐AKT‐mTOR pathway in femoral tissue (Figure 7F). To confirm whether RAPA‐induced mitophagy contributed to an improved osteogenic microenvironment, mitophagy levels in the femur were assessed using IF staining. ZDPR significantly promoted the co‐localization of LC3B and Tom20 (Figure 7G; Figure S7F, Supporting Information) and decreased SQSTM1 accumulation in F4/80^+^ macrophages, but not in CD73^+^ mesenchymal stem cells (Figure S7E,G, Supporting Information). Moreover, ZDPR lowered the expression of pro IL‐1β, and IL‐1β in the femur (Figure S7D, Supporting Information) and enhanced anti‐inflammatory polarization (Figure 7H; Figure S7H, Supporting Information), suggesting an improved osteogenic microenvironment. These findings underscore the role of ZDPR in preventing osteoporosis by inhibiting osteoclast activity and promoting mitophagy.

**Figure 8:**
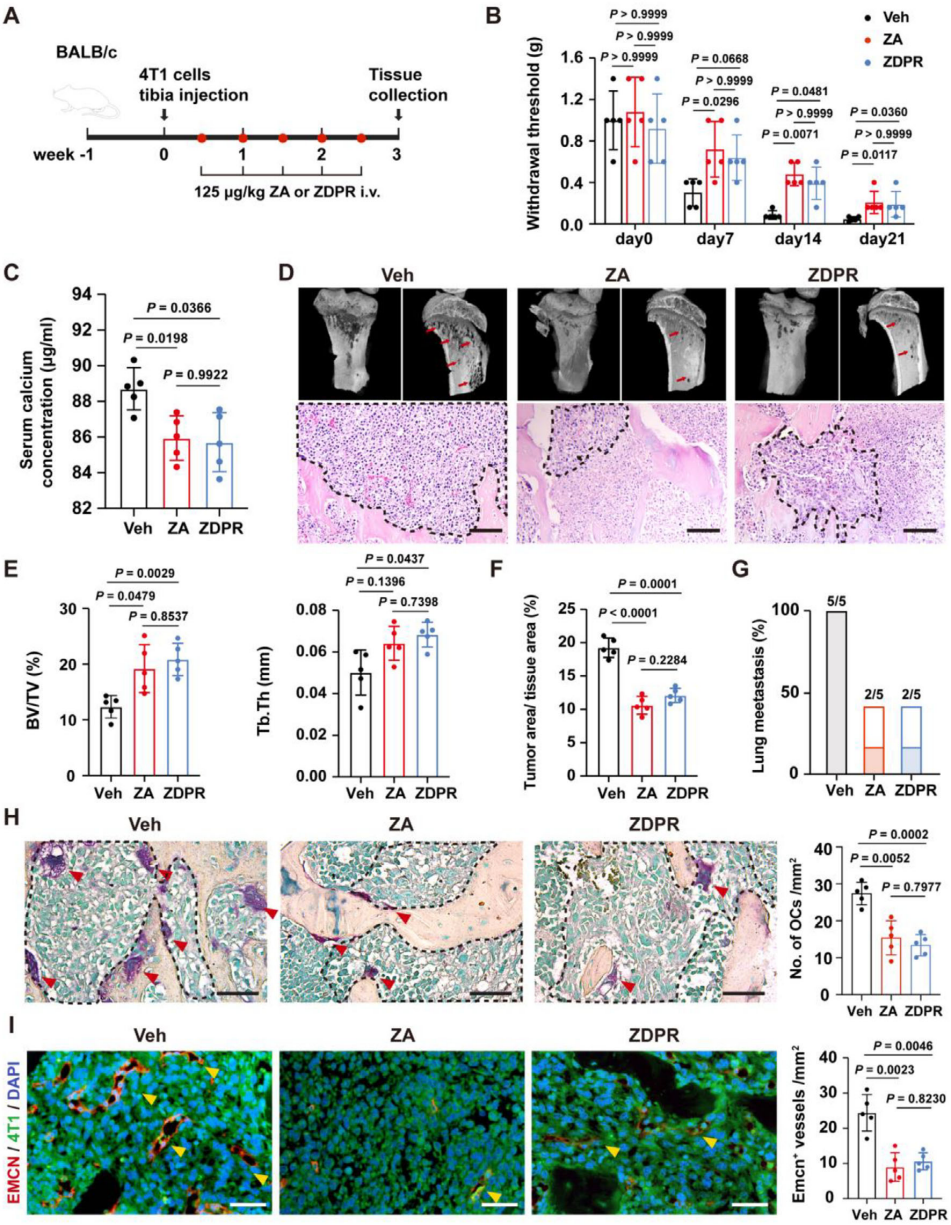
ZDPR protects against malignant tumor‐associated osteolysis. A) Schematic depicting mice breast cancer osteolysis model. B) Von Frey test measuring cancer‐induced mechanical allodynia in mice treated with vehicle (*n* = 5), ZA (*n* = 5), or ZDPR (*n* = 5). C) Serum calcium concentration of mice treated with vehicle, ZA, or ZDPR at endpoint. D) Representative Micro‐CT and H&E‐stained images of the tibia. Dashed areas show tumor lesions in the bone marrow. Scale bars: 100 µm. E,F) Panels show the quantitative measurements of bone volume/tissue volume (BV/TV), trabecular thickness (Tb.Th), and tumor area/tissue area (*n* = 5). G) Panel shows secondary lung metastasis in vehicle, ZA, or ZDPR groups at endpoint. H) Representative TRAP‐stained images of the tibia. Dashed areas show tumor lesions in the bone marrow, with red arrows indicating osteoclasts. Scale bars: 50 µm. I) Immunofluorescence staining detecting EMCN^+^ blood vessels in the bone lesion area, with yellow arrows indicating EMCN^+^ vessels. Scale bars: 50 µm. Results are representative of at least three independent experiments performed. Panels show the quantitative measurements of numbers of osteoclasts (H, *n* = 5), and EMCN^+^ blood vessels (I, *n* = 5). Statistical significance was examined by Mann‐Whitney *U*‐test (B), a two‐sided one‐way ANOVA, or Welch ANOVA (C,E–I).

To further evaluate the therapeutic potential of ZDPR against malignant tumor‐associated osteolytic destruction, murine models were established via intraosseous injection of 4T1 murine mammary carcinoma cells (**Figure** **8**A). Bone pain, a common manifestation of malignant tumor‐associated osteolysis and typically alleviated by ZA,^[^ ^43^, ^44^ ^]^ was examined, revealing that both ZA and ZDPR treatments elevated the pain response threshold in tumor‐bearing mice (Figure 8B). The ZA and ZDPR groups also displayed significantly lower serum calcium levels than the vehicle group (Figure 8C). While the vehicle group exhibited severe osteolytic bone defects and cortical bone discontinuity, both treatment groups showed reduced tumor nests, less extensive bone destruction (Figure 8D–F), and a lower incidence of secondary lung metastasis (Figure 8G, histological data not shown). Furthermore, ZDPR and ZA suppressed osteoclast differentiation and trabecular bone degradation in the tumor‐infiltrated regions (Figure 8H). IF staining revealed a decrease in EMCN^+^ blood vessels within the bone lesions (Figure 8I). Collectively, these findings demonstrate that ZDPR exhibits potent therapeutic efficacy in mitigating malignant tumor‐associated osteolytic destruction.

## 3 Discussion

**Figure 9:**
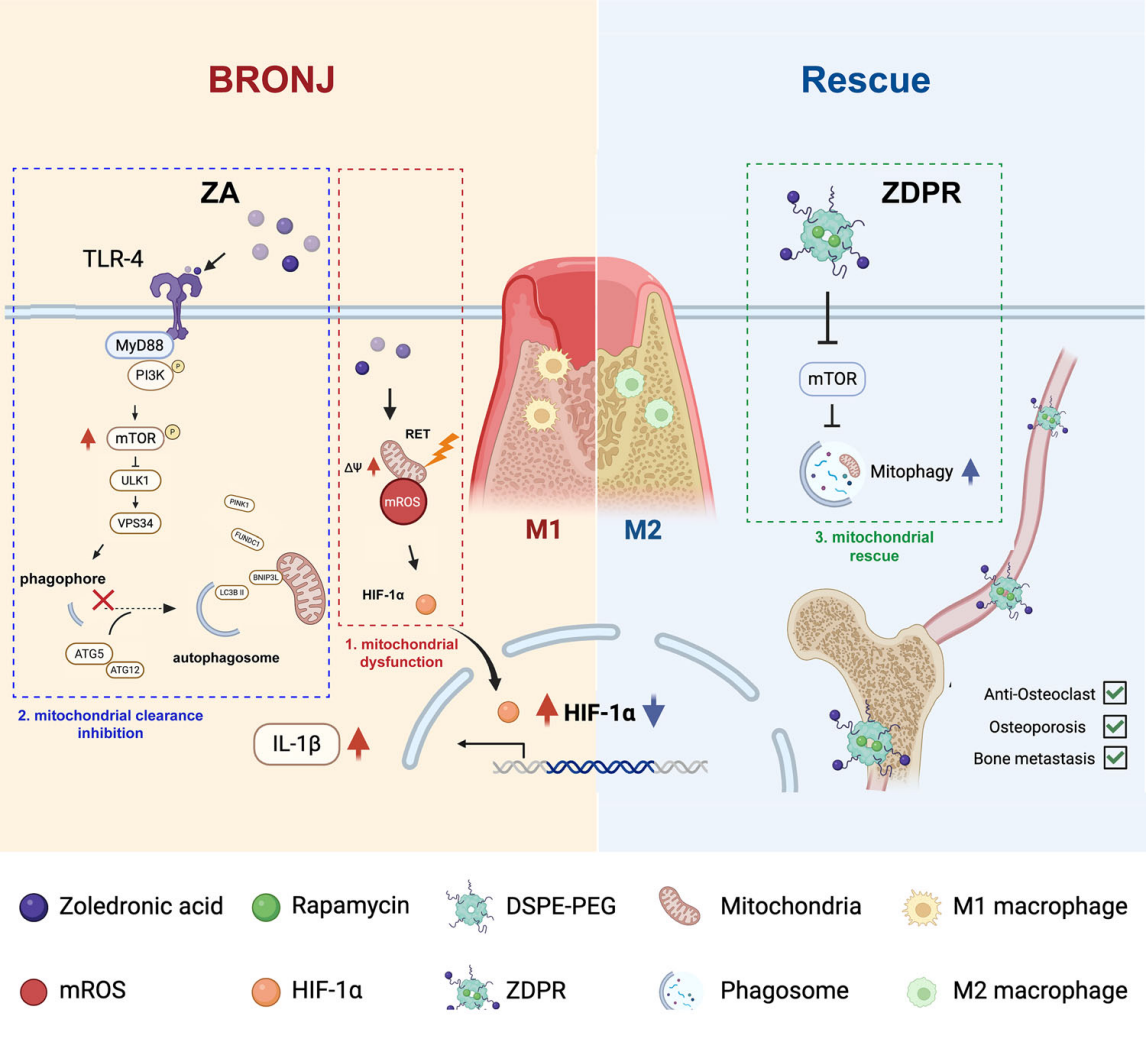
Schematic model showing the suggested mechanism that ZA induces classical activation of macrophages by impairing mitochondrial biofunction and inhibiting mitochondrial clearance to contribute to the pathological process of BRONJ. RAPA‐loaded nanoparticles ZDPR has shown potential in alleviating BRONJ lesions as well as treating osteoporosis or osteolytic bone metastases. Created with BioRender.com.

Emerging evidence indicates that dysregulated macrophage activation contributes to the inflammatory process of BRONJ, although the underlying mechanisms remain unclear. In this study, we demonstrate that ZA impairs mitochondrial bioenergetic function, leading to increased mtROS production and pro‐inflammatory polarization. Furthermore, ZA suppresses autophagy through the TLR4‐MyD88/PI3K‐AKT‐mTOR pathway, disrupting mitochondrial clearance and sustaining mtROS/HIF‐1α signaling. These findings provide new insights into the pathogenesis of BRONJ and suggest potential preventive and therapeutic strategies for anti‐resorptive therapy (**Figure** **9**).

Several hypotheses have been proposed to explain the mechanisms underlying BRONJ development, including impaired bone turnover, reduced angiogenesis, infection, altered immune responses, and soft tissue toxicity.^[^ ^45^ ^]^ Suppression of osteoclast activity represents the central pathological trigger, as similar clinical phenotypes are induced by mechanically distinct anti‐osteoclast agents, such as BPs or denosumab.^[^ ^46^ ^]^ Nevertheless, the high susceptibility of the jawbone to BRONJ is now widely attributed to the unique microbial environment of the oral cavity. Inhibition of osteoclast function decreases bone resorption, facilitating microbial colonization and biofilm formation, which subsequently exacerbate tissue toxicity and bone necrosis.^[^ ^47^, ^48^, ^49^ ^]^ M1 macrophage polarization has been reported in BRONJ patients,^[^ ^50^ ^]^ and our findings, consistent with previous animal studies, emphasize the crucial role of macrophage polarization abnormalities in disease progression. Thus, infections and macrophage dysregulation remain pivotal factors in understanding the pathology of BRONJ.

Dysregulated macrophage polarization displays comparable features across various inflammatory diseases. During the early phase of wound healing, macrophages acquire a pro‐inflammatory phenotype through mtROS production and HIF‐1α stabilization, which support microbial defense and tissue vascularization.^[^ ^51^ ^]^ However, prolonged or sustained activation leads to tissue damage. Evidence suggests that mtROS/HIF‐1α‐mediated aerobic glycolysis is a hallmark of activated macrophages.^[^ ^32^, ^52^, ^53^, ^54^ ^]^ Consistent with these findings, we observed altered mitochondrial bioenergetics, accumulation of intracellular mtROS, and increased HIF‐1α expression while following ZA treatment. Interestingly, ZA inhibited mitochondrial respiration while enhancing ΔΨm and mtROS production. Recent research has shown that activated macrophages generate superoxide via reverse electron transport in complex I while maintaining reduced CoQ levels.^[^ ^31^, ^55^ ^]^ Villalba et al. first demonstrated that specific inhibition of FDPS and the mevalonate pathway by ZA reduces the CoQ_10_ pool in the mouse liver,^[^ ^56^ ^]^ suggesting that ZA may disrupt mitochondrial complex function and drive reverse electron transport, leading to the observed effects. During this process, the failure to eliminate mtROS through efficient mitochondrial clearance serves as a key trigger for inflammatory signaling cascades. Considering the importance of mitophagy in maintaining mitochondrial quality and controlling oxidative stress, our study aimed to elucidate the relationship between ZA‐induced mtROS production and mitophagy inhibition during macrophage metabolic reprogramming.

Defective mitophagy in macrophages has been linked to several inflammatory disorders. Owing to their diverse energy and functional requirements, macrophages tend to shift toward the pro‐inflammatory phenotype when mitophagic activity declines.^[^ ^25^, ^57^ ^]^ The classical mitophagy pathway involves sequential stages, including phagophore initiation, mitophagosome formation, and subsequent maturation into mitolysosomes.^[^ ^58^ ^]^ Disruption of any of these steps disturbs mitochondrial homeostasis and has been associated with numerous human diseases.^[^ ^59^, ^60^ ^]^ Therefore, identifying the specific inhibitory targets of ZA within the mitophagy process is essential. Initially, we observed elevated ΔΨm and impaired recruitment of PINK1/Parkin to the mitochondria after 6 h of ZA treatment. This finding suggests that early mitophagy inhibition may result from impaired PINK1 recruitment, as PINK1 degradation typically occurs at high ΔΨm.^[^ ^61^, ^62^ ^]^ However, we discovered an apparent paradox: CCCP‐induced loss of ΔΨm and increased PINK1 expression did not promote lysosomal degradation of damaged mitochondria. Further bioinformatics and immunoblot analyses revealed enrichment of the PI3K‐AKT‐mTOR signaling pathway. mTOR signaling is widely recognized as a central regulator of conventional autophagy and influences mitochondria clearance by modulating common downstream effectors. Under nutrient‐replete conditions, the nutrient‐sensing mTORC1 phosphorylates the key regulator of lysosomal biogenesis TFEB to inhibit lysosomal biogenesis.^[^ ^63^ ^]^ Liver‐specific deletion of TFEB increased translocation of mTOR into lysosomes, resulting in increased mTOR activation and defected lysosome function and autophagy.^[^ ^64^ ^]^ In this study, we showed that ZA not only suppressed mitophagy initiation but also impaired downstream autophagic flux through mTOR activation, thereby hindering mitochondrial fusion with lysosomes and lysosomal biogenesis. The mTOR‐mediated metabolic switch that occurs during macrophage phenotype conversion is also essential for the execution of pro‐inflammatory functions.^[^ ^65^ ^]^ Conversely, inhibition of mTOR promotes mitophagy, thereby facilitating anti‐inflammatory polarization.^[^ ^66^ ^]^ Consistent with this, our findings provide new evidence that ZA inhibits mTOR through the TLR4‐MyD88/PI3K‐AKT signaling pathway, ultimately driving macrophage pro‐inflammatory polarization. In addition, mTOR‐mediated autophagy is central in the proliferation, differentiation, migration, and function of osteoclasts.^[^ ^67^ ^]^ ROS/TFEB axis‐induced autophagy in osteoclasts contributes to bone homeostasis and bone resorption.^[^ ^68^ ^]^ Therefore, it will be important for future studies to elucidate the role of ZA in regulating osteoclast function through its effects on mTOR signaling, V‐ATPase activity, and lysosomal integrity.

The emergence of biomaterials and pharmacological agents targeting mitophagy has positioned this process as a promising therapeutic strategy for various diseases. For example, Interleukin‐10–loaded extracellular vesicles suppress mTOR to promote mitophagy and prevent acute kidney injury.^[^ ^69^ ^]^ Similarly, mTOR inhibition by RAPA mitigates mitochondrial myopathies,^[^ ^70^ ^]^ while RAPA‐loaded functionalized nanoparticles have been shown to modulate synovial macrophage mitophagy to alleviate osteoarthritis.^[^ ^71^ ^]^ Collectively, these findings suggest that mTOR serves as a potential therapeutic target in inflammatory conditions through the activation of autophagy and mitophagy. Building on the observation of impaired mitochondrial function in BRONJ pathology, we strategically utilized DSPE‐PEG_2000_‐ZA as a bone‐targeting carrier to deliver the mitophagy inducer RAPA. This represents the first application of DSPE‐PEG_2000_‐NH_2_ for the co‐delivery of ZA and RAPA. Our results demonstrate that ZDPR effectively prevents BRONJ and exhibits a favorable safety profile as a rapamycin‐loaded nanoparticle. Furthermore, ZDPR nanoparticles have dual therapeutic potential—treating both osteoporosis and osteolytic lesions associated with malignant bone metastases. Given that mitophagy dysfunction and oxidative stress are common features of osteoporotic bone loss,^[^ ^8^, ^72^ ^]^ and that RAPA derivatives have been approved for breast cancer chemotherapy by targeting mTOR to inhibit tumor cell growth and improve patient survival,^[^ ^73^, ^74^ ^]^ we evaluated the efficacy of ZDPR in both OVX and bone metastasis models. ZDPR not only exerted an osteoclast inhibitory effect comparable to ZA but also significantly enhanced the osteogenic microenvironment through mitophagy activation. Nevertheless, considering the complex mechanisms underlying antitumor responses, further studies are warranted to fully explore the potential of ZDPR and mitophagy activation in the treatment of diverse malignancies. Future investigations should be designed to validate and expand upon these findings.

## 4 Experimental Section

### Mice

Mice were maintained under a 14/10 h light/dark cycle, housed in groups of four to five per standard cage at 22–25 °C, and provided with unrestricted access to standard rodent chow and water in the Animal Research Center of Nanjing Medical University. Six‐week‐old female wild‐type (WT) C57BL/6 and BALB/c mice were obtained from Nanjing Medical University. *Lyz2cre* mice (Cat# N000056) were obtained from the Model Animal Research Center of Nanjing University, and *Atg5^f/f^* mice (JAX# 008208) were purchased from the Jackson Laboratory. To achieve *Lyz2*‐lineage–specifically, knockout of *Atg5*, 3‐month‐old *Lyz2cre* males were mated with age‐matched *Atg5^f/f^* females to produce *Lyz2cre; Atg5^f/+^* offspring. Male *Lyz2cre; Atg5^f/+^* mice were subsequently backcrossed with *Atg5^f/f^* females to generate *Lyz2cre; Atg5^f/f^* homozygous cKO mice, while *Atg5^f/f^* littermates were used as floxed controls. All experimental protocols were approved by the Ethics Committee of the School of Stomatology, Nanjing Medical University (approval nos. IACUC‐1805006 and IACUC‐2501027). All animal procedures followed the institutional guidelines of the Animal Care Committee of Nanjing Medical University.

### Animal Experiments

In the BRONJ mouse model, 8‐week‐old female C57BL/6J mice (*n = *5 per group), *Atg5^f/f^* mice (*n = *5 per group), or *Lyz2cre; Atg5^f/f^* mice (*n = *5 per group) were intravenously injected with 125 µg kg^−1^ ZA (Apexbio, A1352) via the tail vein according to established procedures.^[^ ^10^, ^19^ ^]^ One week after ZA administration, the right maxillary molars were extracted under general anesthesia using isoflurane inhalation. Two additional ZA doses were administered at weekly intervals for two consecutive weeks. For BRONJ prevention, ZDPR solution (125 µg kg^−1^) was intravenously administered following the same dosing schedule. At the experimental endpoint, intraoral photographs of the TES were captured, and the maxillae, along with major organs, were collected for further analysis.

### Histology Analysis

For histology evaluation, hematoxylin and eosin (H&E) and TRAP staining were performed on 4 µm–thick sections derived from formalin‐fixed, paraffin‐embedded tissues. Sections were deparaffinized in xylene and rehydrated through graded ethanol to distilled water. The slides were stained using hematoxylin and eosin or a TRAP staining kit (Millipore Sigma, 387A‐1KT), following the manufacturer's protocols. For quantification of osteonecrosis area, H&E‐stained sections were scanned using an Olympus scanning system VS200 for measurement according to widely accepted protocols in BRONJ studies.^[^ ^75^ ^]^ An area of interest located within 1.2 mm of the number 2 molar was selected. The osteonecrosis area defined as any 1000‐µm^2^ area that contains three or more empty lacunae was marked for analyses.

### Micro‐CT Analysis

Micro‐CT data were acquired using a SkyScan 1176 system (Kontich, Belgium). The microarchitectural properties of the TES, femur, and tibia were scanned at a high resolution of 18 µm, using an energy setting of 50 kV and 456 µA. The regions of interest (ROI) were defined as the tooth extraction socket, distal femurs, and proximal tibia. Bone morphology, 3D bone reconstructions, bone mineral density (BMD; mg cm^−3^), trabecular bone volume/tissue volume ratio (BV/TV; %), trabecular thickness (Tb.Th; mm), trabecular number (Tb.N; 1/mm), and trabecular separation (Tb.Sp; mm) were analyzed using CTVox v.3.2 and CTAn v.1.13.8.1 software (SkyScan).

### Cell Culture

Murine BMDMs were isolated from the femurs of C57BL/6 mice as previously described.^[^ ^19^ ^]^ Briefly, bone marrow cells were flushed from the femurs and collected in DMEM containing 2% FBS and 1% penicillin‐streptomycin. Erythrocytes and other non‐adherent cells were removed using RBC lysis buffer (Beyotime, C3702). The remaining cells were cultured in DMEM (Gibco, 11995500BT) containing 10% FBS, 1% penicillin‐streptomycin, and 10 ng ml^−1^ recombinant murine macrophage colony‐stimulating factor (M‐CSF; Pepro Tech, 315‐02) for 7 days to induce macrophage differentiation. For polarization, macrophages were stimulated with either 100 ng mL^−1^ LPS or 20 ng mL^−1^ IL‐4. For the osteoclast inhibition assay, BMDMs were cultured in DMEM supplemented with 10% FBS, 1% penicillin‐streptomycin, 10 ng mL^−1^ M‐CSF, and 20 ng mL^−1^ recombinant murine receptor activator of NF‐κB Ligand (RANKL; Pepro Tech, 315‐11) to induce osteoclast differentiation. Cells were then treated with vehicle, 10 µm ZA, or 10 µm ZDPR to assess anti‐osteoclastic activity.

### RNA Sequencing Analysis

Total RNA was extracted from BMDMs treated with or without ZA using TRIzol reagent (Thermo Fisher Scientific, 15596018CN) according to the manufacturer's instructions. RNA purity and concentration were determined using a NanoDrop 2000 spectrophotometer (Thermo Fisher Scientific), and RNA integrity was assessed using an Agilent 2100 Bioanalyzer (Agilent Technologies). RNA libraries were prepared with the VAHTS Universal V10 RNA‐seq Library Prep Kit (Premixed Version) following the manufacturer's protocol. Sequencing was performed on an Illumina Novaseq 6000 platform to generate 150 bp paired‐end reads. Transcriptome sequencing and subsequent analyses were conducted by OE Biotechnology. The raw sequencing data used in this study are available in the NCBI GEO under accession number GSE306512.

### Activators and Inhibitors

Baf A1 (Abcam, ab120497) was applied as a selective vacuolar (V)‐ATPase inhibitor at a working concentration of 10 nm. RAPA (MCE, HY‐10219) served as an autophagy activator at 50 nm, whereas 3‐MA (MCE, HY‐19312) functioned as an inhibitor at 5 mm. TAK‐242 (MCE; HY‐11109) selectively inhibited TLR4 at 10 nm. NAC (Aladdin, N755681) was used as an antioxidant at 500 µm. CCCP (Beyotime, C2006) acted as a mitochondrial uncoupler at 10 µm. All activators and inhibitors were added to the conditioned medium for cell incubation.

### Mitochondrial Protein Isolation

Mitochondria were isolated to assess the recruitment of specific proteins using a mitochondria isolation kit (Beyotime, China). BMDMs were harvested and resuspended in 1 mL of separation reagent before being homogenized with a glass homogenizer. The homogenates were centrifuged at 600 g for 10 min at 4 °C, and the supernatant was further centrifuged at 11 000 g for 10 min to pellet mitochondria. The precipitates were lysed to obtain mitochondrial proteins.

### Western Blot Analysis

For western blotting, total and mitochondrial proteins were extracted using RIPA buffer (Beyotime, P0013B) containing PMSF (Beyotime, C3601‐5) and a phosphatase inhibitor cocktail (Apexbio, K1015). Proteins were separated on 8–12% SDS‐PAGE gels and transferred to 0.45 µm PVDF membranes (Millipore, IPVH00010). After blocking for 2 h (Beyotime, P0216), membranes were incubated overnight at 4 °C with primary antibodies. Following three washes with Tris‐buffered saline containing Tween‐20, membranes were incubated with secondary antibodies for 1 h at room temperature and visualized using ECL detection (Millipore, WBULP‐100ML). Details of the antibodies are listed in Table S1 (Supporting Information). Protein band intensities were analyzed using ImageJ software, and relative protein levels were expressed as ratios to β‐actin and normalized to control values.

### Flow Cytometry Analysis

To evaluate macrophage polarization in the TES, mouse jawbones were harvested and gently ground into single‐cell suspensions according to the previous protocols.^[^ ^76^ ^]^ The cells were washed with PBS and stained with Fixable Viability Dye eFluor 506 (Invitrogen, 65‐0866‐14), APC‐eFlour 780 Anti‐Mouse CD45 (Invitrogen, 47‐0451‐82), PerCP/Cyanine5.5 Anti‐Mouse CD11b (Elabscience, E‐AB‐F1081J), Brilliant Violet 605 Anti‐Mouse F4/80 (Biolegend, 123133), PE Anti‐Mouse CD86 (BD Biosciences, 561963), and FITC Anti‐Mouse CD206 (Biolegend, 141703) antibodies. To detect mitochondrial density in BMDMs, cells were treated as indicated and stained with Mito‐Tracker Green (Beyotime, China) at 37 °C for 30 min. Flow cytometry was conducted using a BD FACSymphony A5 flow cytometer (BD Biosciences), and data were analyzed with FlowJo v.10 software.

### mt‐Keima Fluorescence Imaging

Mitophagy events in BMDMs were visualized using mt‐Keima fluorescence imaging with a Leica Stellaris STED confocal microscope. After transfection with mt‐Keima, cells were treated as specified and seeded onto glass‐bottom dishes. They were incubated in a live‐cell imaging solution (Invitrogen, A59688DJ) and imaged sequentially at 488 nm (neutral pH Keima) and 561 nm (acidic pH Keima). The mt‐Keima sequences are listed in Table S2 (Supporting Information).

### Immunofluorescence and Immunochemistry Staining

For co‐localization detection, polarization‐related proteins, or HIF‐1α detection, BMDMs were seeded on coverslips for 24 h at 37 °C before receiving the indicated treatments. The cells were fixed with 4% PFA, permeabilized with 1% Triton X‐100, and incubated with the primary antibodies listed in Table S1 (Supporting Information) overnight at 4 °C after blocking with goat serum. The next day, cells were incubated with Cy3‐ or FITC‐labeled secondary IgG for 1 h. Finally, coverslips were counterstained with DAPI for 1 min and visualized using a Leica Stellaris STED confocal microscope. For tissue immunostaining, the TES, femur, or tibia was fixed in 4% PFA for 48 h and dehydrated in 30% sucrose for 24 h before being embedded in OCT to prepare 6‐µm‐thick cryosections. Sections were permeabilized with 0.1% Triton X‐100 for 5 min, blocked with goat serum, and stained with appropriate antibodies for fluorescence microscopy. For immunochemical staining of the mouse tibia, paraffin‐embedded sections were deparaffinized, rehydrated, subjected to sodium citrate heat‐induced antigen retrieval for 15 min, and blocked with goat serum for 30 min at room temperature. Sections were then incubated with primary and secondary antibodies (Table S1, Supporting Information) for 3,3′‐Diaminobenzidine (DAB) and hematoxylin staining.

### Transmission Electron Microscopy Scanning

The TEM was conducted as previously described. Briefly, BMDMs from each treatment group were collected, the culture medium was removed, and they were gently washed with PBS. The cells were then digested with trypsin and centrifuged to discard the supernatant. Subsequently, they were fixed in an electron microscopy fixation solution (Solarbio, P1126) and stored at 4 °C. Sample preparation was performed at the Analysis and Testing Center of Nanjing Medical University, and images were acquired using a transmission electron microscope (JEOL, JEM‐1010).

### Mitochondrial ΔΨm and ROS Production

For assessing mitochondrial ΔΨm detection, cells were cultured on glass coverslips and stained with either the JC‐1 kit (Beyotime, C2005) or 50 nm tetramethylrhodamine (TMRM) (Abcam, ab274305). The coverslips were then mounted in cold PBS and directly visualized using a confocal fluorescence microscope (Zeiss, CLSM710). For flow cytometry analysis, JC‐1‐stained cells were washed, resuspended in PBS, and analyzed. For ROS detection, BMDMs were incubated for 20 min at 37 °C with 2.5 µm MitoSOX red (Invitrogen, M36008) for mtROS or 5 µm DCFH‐DA (Beyotime, S0033S) for total ROS after stimulation. Cells were analyzed using either flow cytometry or a microplate reader (Molecular Devices, SpectraMax M5).

### Statistics and Reproducibility

All experiments were independently repeated at least three times. Data normality was tested using the Shapiro–Wilk test. Statistical analyses were performed using two‐tailed Student's *t*‐test, two‐sided unpaired Welch's *t*‐tests, Mann‐Whitney U‐test, or one‐way analysis of variance (ANOVA) or Welch ANOVA with Tukey's multiple comparison test. Sample sizes (*n*) were determined based on adequate biological or technical replicates and are specified in each figure legend. Statistical significance was set at *p* <0.05 (^*^), with exact *p*‐values provided in the figures. Graphs and plots were generated using GraphPad Prism software (v9.3.0 for MacOS).

## Conflict of Interest

The authors declare no conflict of interest.

## Author Contributions

H.Z. and X.S. contributed equally to this work. H.J. was responsible for funding acquisition, conceptualization, and designing of the research, and supervised the experiments. H.Z. performed experiments, analyzed data, and drafted the manuscript. X.S. assisted H.Z. with animal studies and data analysis. H.L. and X.Y. performed the bioinformatics analysis. M.C. and X.L. synthesized and characterized ZDPR nanoparticles. Z.L., S.G., and R.X. contributed to experimental design and validation. H.J. and X.L. revised the manuscript. All authors reviewed and approved the final manuscript.

## Supporting information



Supporting Information

Supporting Information

Supplementary Tables

## Data Availability

The data that support the findings of this study are available from the corresponding author upon reasonable request.
