# Supplementary material for: Rescuing Mitochondrial Dysfunction in Macrophages Prevents Osteonecrosis of the Jaw in Anti‐Resorptive Therapy
Source: Adv Sci (Weinh). 2025 Dec 7;13(11):e17586. doi: 10.1002/advs.202517586 (PMC12931161; doi:10.1002/advs.202517586)
Supplement: Supplementary file 1 — Supporting Information [file ADVS-13-e17586-s001.docx]

Supporting Information

Rescuing Mitochondrial Dysfunction in Macrophages Prevents Osteonecrosis of the Jaw in Anti-resorptive Therapy

Hang Zhang, Xin Shen, Haiyang Liu, Xinxi Yuan, Mumin Cao, Xuepeng Lv, Ziji Ling, Songsong Guo, Rongyao Xu, Xiang Li, Hongbing Jiang*

**Supplementary methods**

***Cell culture***

Lucifer-labeled (zqxzbio, LZQ0016) or GFP-labeled (zqxzbio, GZQ0017) 4T1 murine mammary tumor cell lines were maintained in RPMI-1640 medium (Gibco, C11875500BT) with 1% penicillin-streptomycin, and 5% FBS.

***Measurement of real-time oxygen consumption rate***

OCR were measured using a Seahorse XFe96 analyzer (Agilent, S7800AR) according to the manufacturer’s instructions. Briefly, 4×10^4^ BMDMs were pre-treated with vehicle solution or 10 μM ZA for 24 hours, plated on the XFe96 cell culture microplate and cultured as indicated. The detection with ATP synthase inhibitor oligomycin and electron-transport chain inhibitor antimycin A/rotenone treatment was entrusted to the Nanjing Medical University Analysis and Testing Center. Data were analyzed using the Wave software.

***Preparation of ZDPR***

A quantity of 100 mg of ZA was dissolved in triethylamine (TEA) and distilled dimethylformamide (DMF). The mixture was transferred to a closed container under a nitrogen atmosphere, and 90 mg of 1,1'-carbonyldiimidazole (CDI) was added to the solution. The reaction mixture was continuously stirred at 60°C in an oil bath for 24 hours. TEA was distilled out in a rotary evaporator, leading to the precipitation of activated ZA, which was obtained by centrifugation. The precipitate was washed twice with methylene chloride to remove CDI and then dried in a rotary evaporator to obtain purified active ZA. Subsequently, 1 g of DSPE-PEG_2000_-NH_2_ (Ruixibio, R-0038) and 22.6 mg of activated ZA were dissolved in dimethyl sulfoxide (DMSO) with TEA and allowed to react in a closed container under a nitrogen atmosphere for 12 hours. The product was purified by column chromatography using methylene chloride as the mobile phase, and DSPE-PEG_2000_-ZA conjugate was obtained by drying in a rotary evaporator. The DSPE-PEG_2000_-ZA conjugate was dissolved in water, and a solution of RAPA in tetrahydrofuran (THF) was added dropwise. THF was then replaced with nitrogen gas, yielding the product RAPA-DSPE_2000_-PEG-ZA. Solution samples were prepared for dynamic light scattering analysis of particle size using a NanoSizer and Zeta potential analyzer (Malvern, ZS90). The samples were dissolved in CDCl3 for analysis by nuclear magnetic resonance spectroscopy (NMR) using a nuclear magnetic resonance spectrometer (Bruker, AVANCE400). The samples were coated on glass slides, vacuum-dried, gold-sputtered, and observed for particle morphology using a scanning electron microscope (SEM) (Hitachi, SU3500).

***Entrapment efficiency and drug loading***

A precise 200 μL sample of rapamycin-loaded ZDPR nanoparticles was placed into an ultrafiltration tube and centrifuged at 10,000 rpm for 1 hour at 4°C. The unencapsulated free rapamycin was separated through the ultrafiltration membrane. A 20 μL aliquot of the free drug solution was then transferred to a 10 mL volumetric flask and diluted to the mark with methanol for analysis. The absorbance of the sample was measured at 278 nm, and the rapamycin concentration was determined from a standard calibration curve and the total amount of unencapsulated free rapamycin in the original solution was calculated. The entrapment efficiency and drug loading of the lipid nanoparticles were then determined using the following formulas:

$$Entrapment efficiency \left( \% \right)=\frac{unencapsulated free rapamycin}{Total mass of rapamycin added}\times100\%$$

$$Drug loading \left( \% \right)=\frac{Mass of encapsulated rapamycin}{\left( Mass of encapsulated rapamycin + Mass of carrier \right)}\times100\%$$

***RAPA drug release***

The in vitro release of rapamycin-loaded ZDPR nanoparticles were evaluated using a reverse dialysis method. A 30 mL volume of PBS was used as the release medium and placed into a 50 mL centrifuge tube. 1 mL of the release medium was added to a dialysis bag, which was then sealed at both ends and immersed in the centrifuge tube. A 200 μL sample of ZDPR nanoparticles (with free drug removed) was added to the release medium. The centrifuge tube was placed on a shaker set to 37°C and 150 rpm. At various time points (1, 2, 4, 8, 12, 24, 36, 48, 60, 72, 96, 120, and 144 hours), 200 μL samples were collected from the dialysis bag. Each sample was transferred to a 5 mL volumetric flask, and methanol was added for subsequent analysis and calculation of the cumulative release rate. After each sampling, 200 μL of fresh release medium was added to the centrifuge tube to maintain a consistent volume.

***CCK-8 assay***

A CCK-8 kit (Apexbio, K1018) was used to assess IC50 of ZDPR. BMDMs were treated with ZDPR at different concentrations for 24 h and then seeded in 96-well plates (3 ×10^3^/well). 10 μl CCK-8 reagent resuspended with 100 μl DMEM was added to each well and then incubated at 37℃ for 2 hours. Absorbance at 450 nm was then measured via microplate reader (Molecular Devices, SpectraMax M5).

***Quantification of Rapamycin Concentration via LC-MS/MS***

Femurs, maxillae, livers, and kidneys were harvested from C57BL/6J mice injected with DSPE-PEG-RAPA or ZDPR nanoparticles and stored at -80 °C until analysis. For sample preparation, 50 μL of tissue homogenate or whole blood was mixed with 200 μL of working solution containing an internal standard (6 ng/mL verapamil in methanol–acetonitrile, 50:50, v/v). The mixture was vortexed for 5 min and subsequently centrifuged at 12,000 rpm and 4 °C for 5 min. A 150 μL aliquot of the supernatant was then transferred to a 96-well injection plate. Rapamycin concentrations in whole blood and tissue homogenates were quantified using liquid chromatography–tandem mass spectrometry (LC–MS/MS). Calibration curves were constructed by spiking blank matrix (blood or tissue homogenate) with known concentrations of rapamycin and the internal standard. Final rapamycin concentrations are expressed as ng/mL for blood and ng/g for tissue homogenates.

***Establishment of osteoporosis and osteolysis models***

In the osteoporosis model, ovariectomies (OVX) were performed on 8-week-old female C57BL/6J mice (n=5 per group) of the OVX, OVX + ZA groups, and OVX + ZDPR groups. Both ovaries were identified adjacent to the kidneys and resected dorsally under anesthesia. Mice in the Sham group went through the same surgical procedure except removing the ovaries. 4 weeks after OVX or Sham operations, a total of 8 doses of ZA (125 μg/kg) or ZDPR (125 μg/kg) were injected for 8 consecutive weeks while mice of Sham and OVX group received vehicle solution. The femurs were harvested at endpoint for analysis.

In the breast cancer bone metastasis model, 8-week-old female BALB/c mice received direct injection of 4T1 cells (5 × 105 cells in 10 μL PBS) into the right tibia with a 23-gauge needle. The mice loaded with tumor were randomly divided into three groups (n = 5): Veh group, ZA group, and ZDPR group. On the fourth day after injection of tumor cells, the different groups of mice were administered with vehicle solution, ZA (125 μg/kg) or ZDPR (125 μg/kg) twice a week via tail vein. The serum, tibias and lungs were harvested at endpoint for analysis.

***Von Frey testing***

For Von Frey testing, mice were confined to individual 5 × 5 cm boxes placed on an elevated wire grid. A blinded experimenter stimulated their hindpaws using a series of von frey filaments with logarithmically increasing stiffness (0.02–2.56 g, Stoelting). Each filament was applied perpendicularly to the central plantar surface. The 50% paw withdrawal threshold was determined using Dixon’s up-down method.

***Serum calcium measurement***

At endpoint of murine breast cancer bone metastasis model, peripheral blood of BALB/c mice were collected and centrifuged at 4000 r/min for 5 minutes to separate the serum. Subsequently, the serum from each experimental group was analyzed for calcium levels using a calcium assay kit (Beyotime, S1063S).

**Supplementary figures and legends**


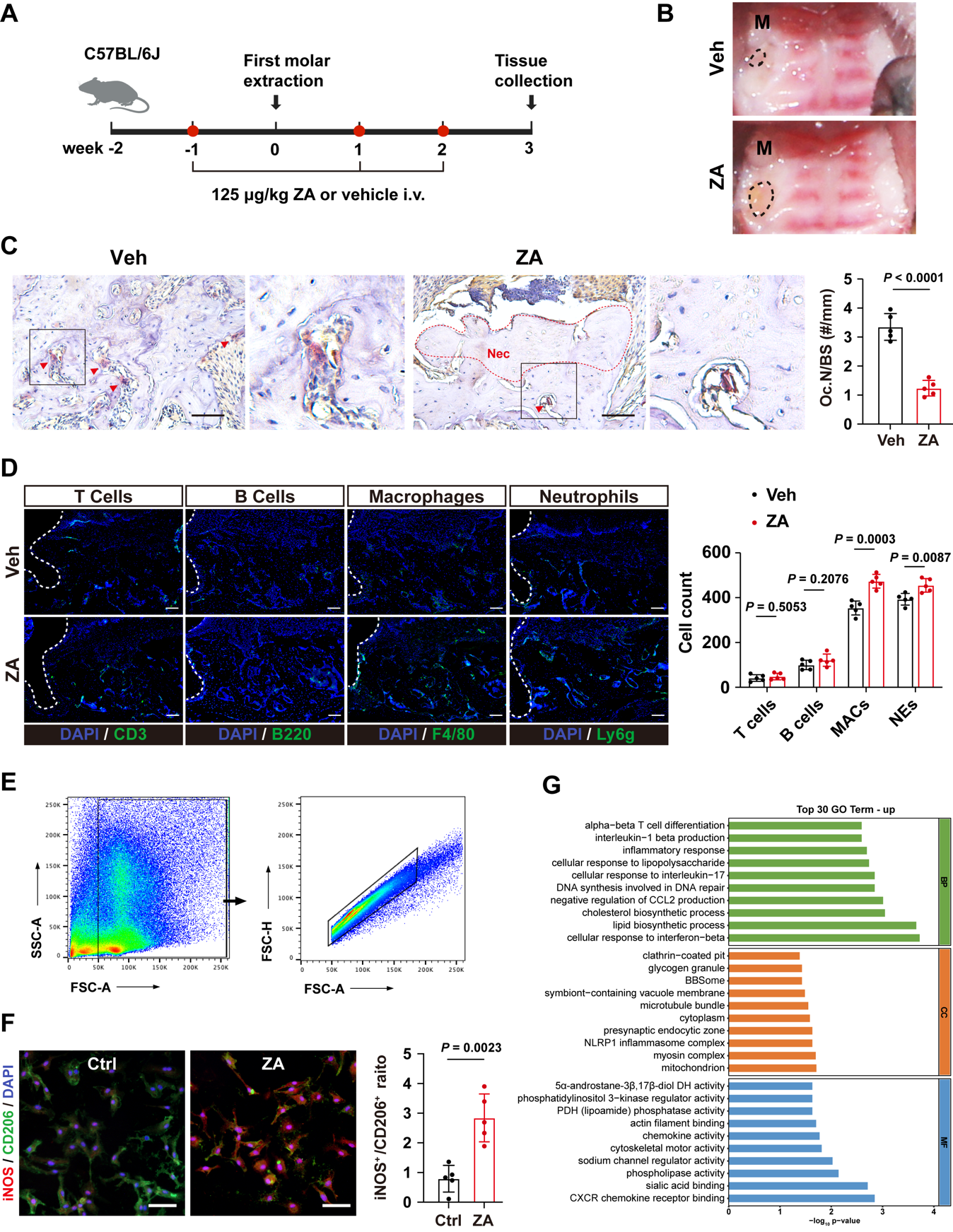


**Figure S1. Effects of ZA on macrophage polarization and mitochondria in BRONJ conditions.** (**A**) Experimental schedule for inducing BRONJ lesions in mice. ZA or vehicle solutions were administrated as indicated. (**B**) Intra-oral photographs of the healing of tooth extraction site (TES) and soft tissue 3 weeks after tooth extraction. Dashed lines indicate the range of wound adjacent to the second molar (M). (**C**) Representative TRAP-stained images of mice TES treated with vehicle solution or ZA. Scale bars: 100 μm. (**D**) Immunofluorescence (IF) imaging of CD3+ cells (T cells), B220+ cells (B cells), F4/80+ cells (macrophages), and Ly6g+ cells (neutrophils) in tooth extraction socket (TES). White dashed lines indicate the root of second molar. Scale bars: 100 μm. (**E**) Gating strategy for jawbone cells. (**F**) Representative images of Ctrl or ZA-treated macrophages stained with M1 marker iNOS or M2 marker CD206. The right panel shows the quantitative measurements of iNOS^+^/ CD206^+^ ratio (*n* = 5). Scale bars: 100 μm. (**G**) Top pathways identified by GO enrichment analysis from differentially expressed genes of BMDMs after ZA treatment. Results are presented as the mean ± SD.


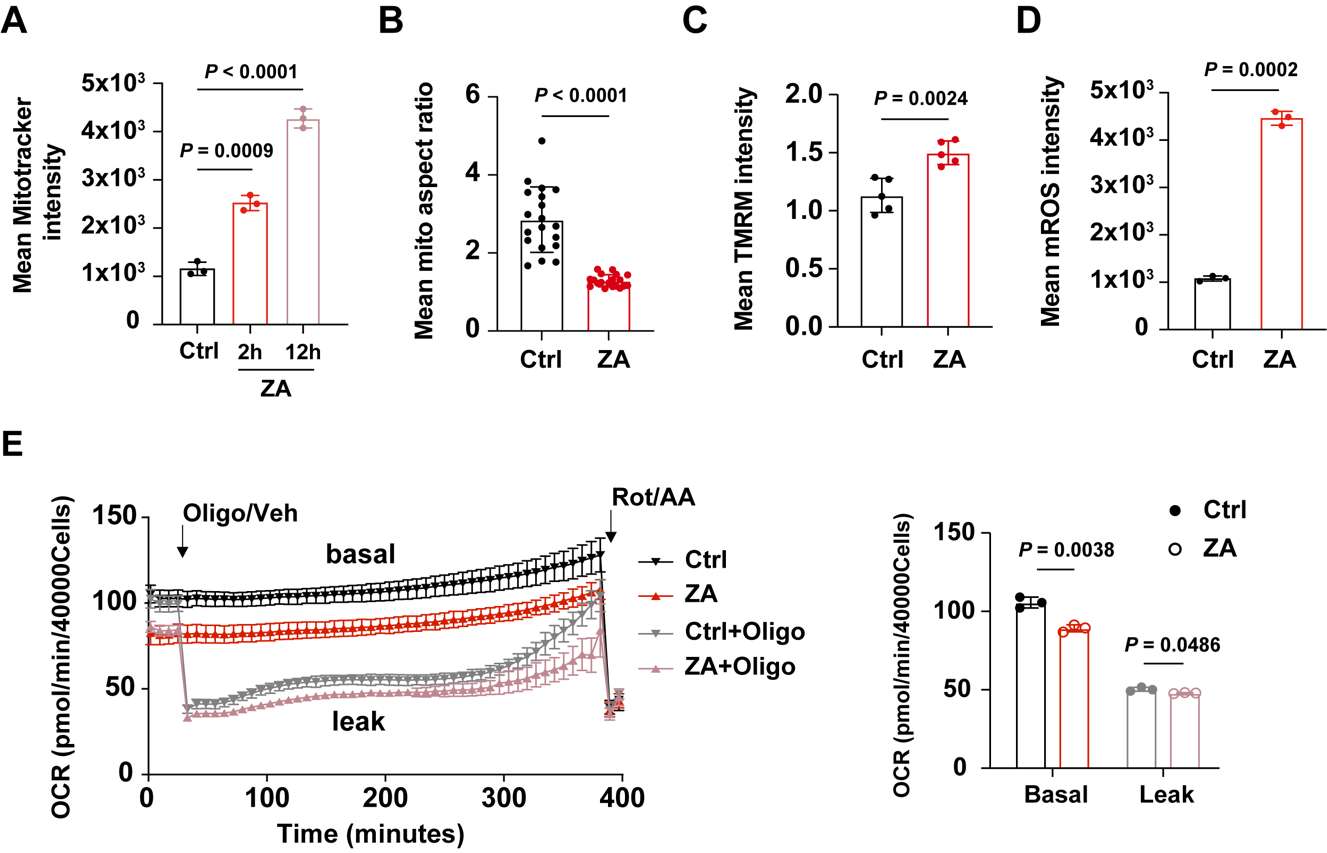


**Figure S2. ZA raises Δψm and promotes HIF-1α expression in BMDMs.** Panels show the quantitative measurements of mean Mitotracker intensity (**A**, *n* = 3), mean mitochondrial aspect ratio (**B**, *n* = 20), mean expression level of TMRM (**C**, *n* = 5), and mean mROS intensity (**D**, *n* = 3). (**E**) Seahorse XFe96 measuring oxygen consumption rate (OCR) of BMDMs after 24 hours of ZA pretreatment. Arrow indicates the addiction of 5 μM Oligomycin (Oligo) and 1 μM antimycin A/rotenone (Rot+AA). Results are presented as the mean ± SD.

**
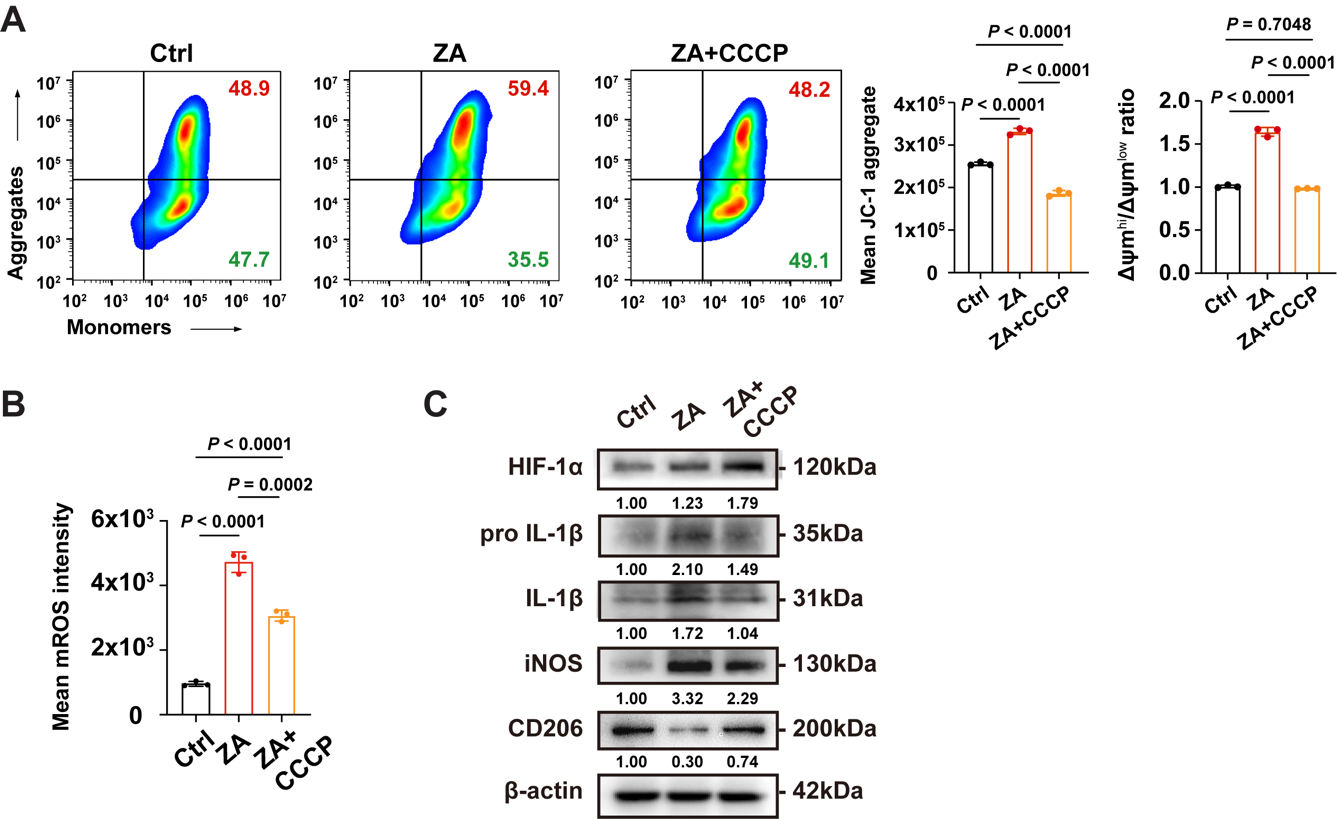
**

**Figure S3. Decrease of Δψm by CCCP does not effectively induce mitophagy in ZA-treated BMDMs.** (**A**) Flow cytometry assessment of mitochondrial membrane potential in BMDMs treated with ZA only, or with ZA+CCCP. The right panels show the quantitative measurements of mean expression level of JC-1 aggregates (*n* = 3) and JC-1 aggregates/monomers ratio (*n* = 3). (**B**) Panels show the quantitative measurements of mean mROS intensity (*n* = 3). (**C**) Westernblot analysis of HIF-1α, pro IL-1β, IL-1β, iNOS, and CD206 in BMDMs with ZA or ZA+CCCP interventions for 24 hours. Densitometric quantifications are indicated beneath the representative blots. Results are presented as the mean ± SD.

**
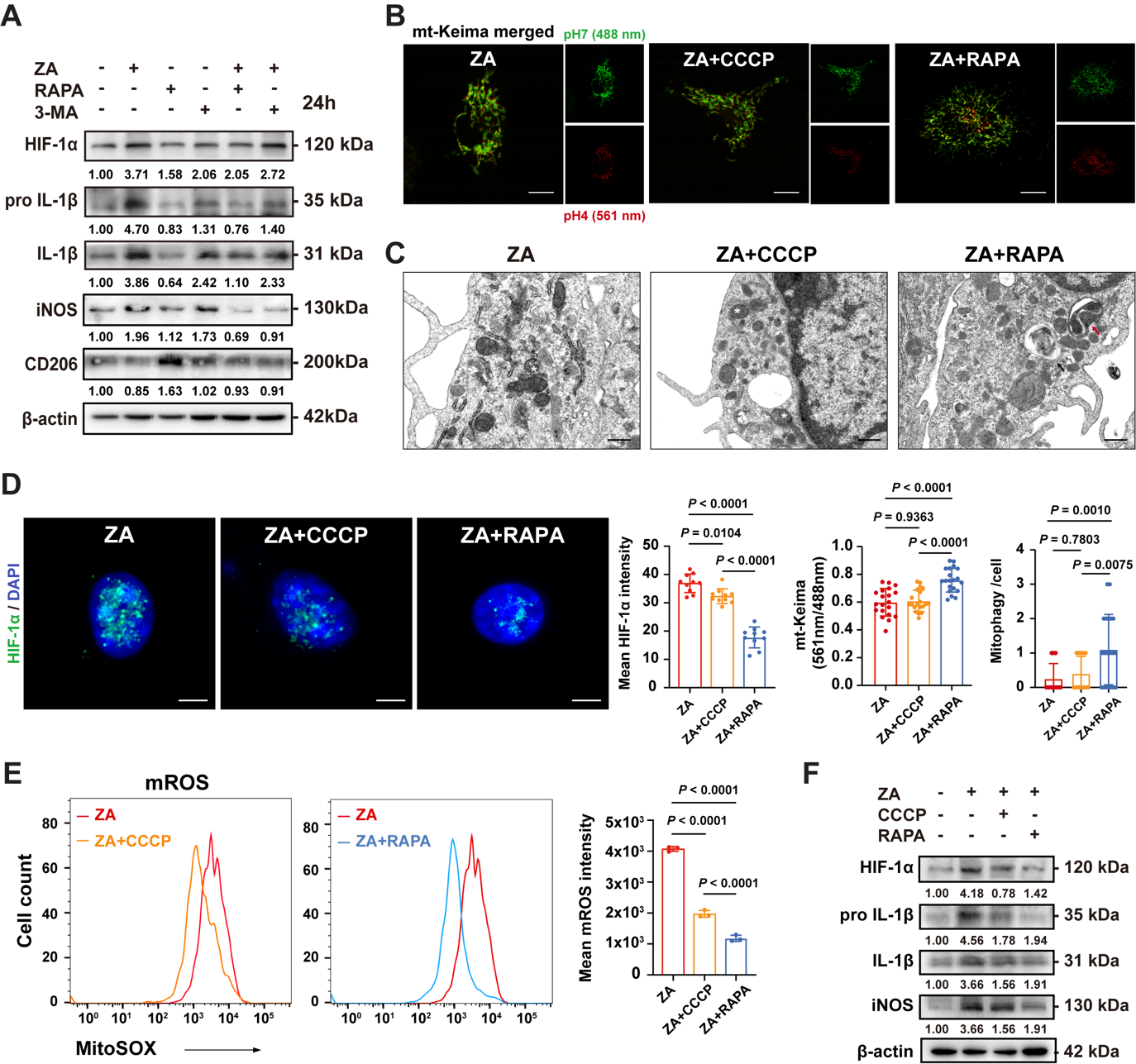
**

**Figure S4. RAPA rescues mitophagy and promotes an anti-inflammatory phenotype of macrophages. (A)** The total protein expression of HIF-1α, pro IL-1β, IL-1β, iNOS, and CD206 in BMDMs with ZA, RAPA, or 3-MA interventions for 24 hours. **(B)** Fluorescence imaging of mitophagy events in BMDMs expressing mt-Keima, and treated with ZA only, with ZA+CCCP, or with ZA+RAPA for 6 hours. The panel below shows quantitative measurements for the ratio of signal excited at 561 nm and 488 nm (*n* = 20). Scale bars: 10 μm. (**C**) Representative TEM images of BMDMs treated with ZA only, with ZA+CCCP, or with ZA+RAPA for 6 hours. Red arrow indicates typical formation of mitophagic vacuoles. White asterisks indicate damaged mitochondria. The panel below shows the quantitative measurements of numbers of mitophagy events per cell (*n* = 20). Scale bars: 500 nm. (**D**) HIF-1α expression in BMDMs treated with ZA only, with ZA+CCCP, or with ZA+RAPA for 24 hours. The right panel shows quantitative measurements of mean HIF-1α expression (*n* = 10). Scale bars: 5 μm. (**E**) Flow cytometry assessment of mitochondrial ROS in BMDMs treated as indicated. The right panel shows quantitative measurements of mean mROS intensity (*n* = 3). (**F**) Westernblot analysis of HIF-1α, pro IL-1β, IL-1β, and iNOS in BMDMs with indicated interventions. Densitometric quantifications are indicated beneath the representative blots. Results are presented as the mean ± SD.


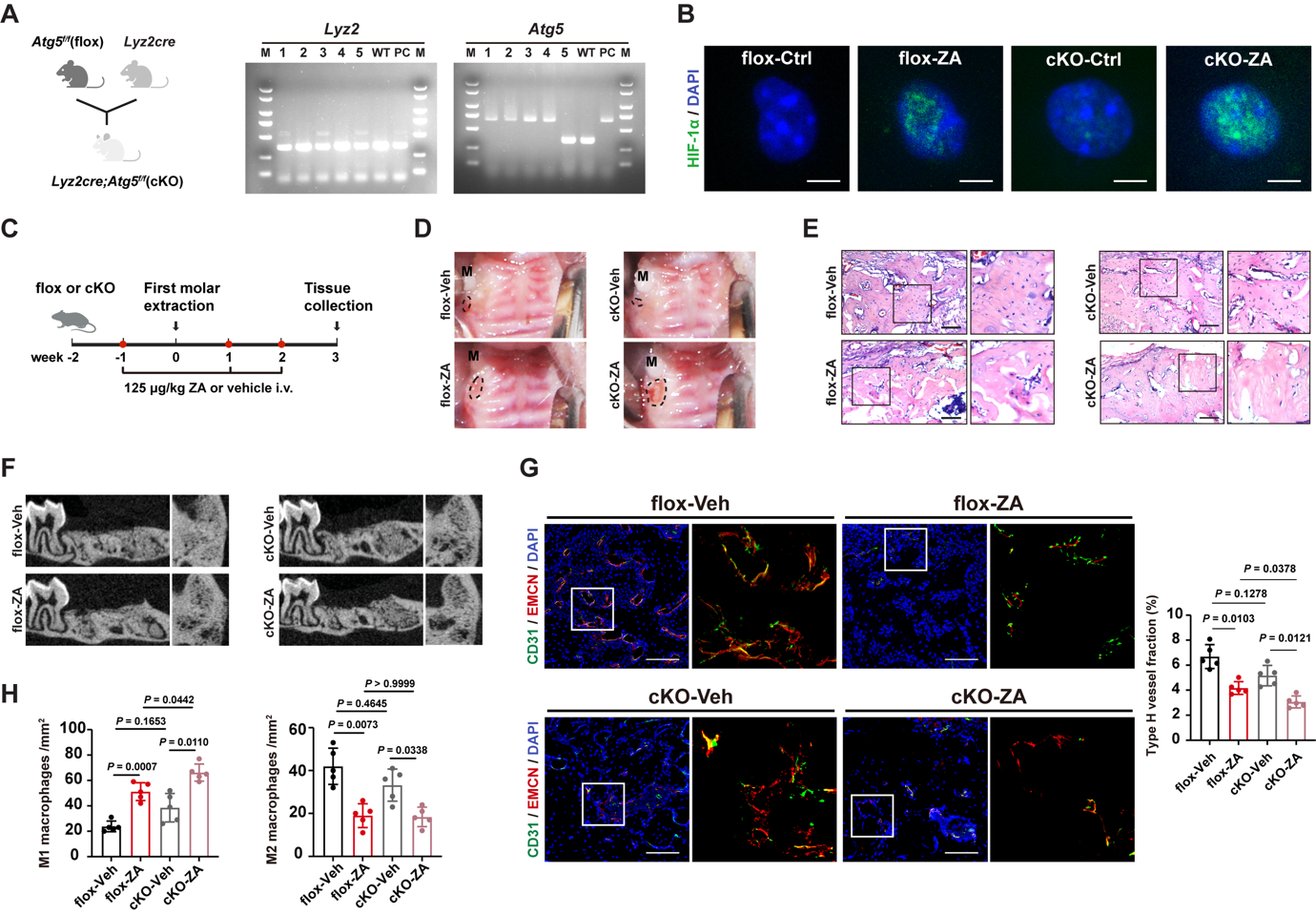


**Figure S5. Deficiency of autophagosome biogenesis induces macrophage pro-inflammatory polarization and aggravates BRONJ.** (**A**) Identification of *Lyz2cre; Atg5^f/f^* mice. (**B**) HIF-1α expression in BMDMs. (**C**) Experimental schedule for inducing BRONJ lesions in flox or cKO mice. ZA or vehicle solutions were administrated as indicated. (**D**) Intra-oral photographs of the healing of TES and soft tissue. Dashed lines mark the range of wound adjacent to the second molar (M). (**E**) Representative H&E-stained images of TES. Scale bars: 100 μm. (**F**) Micro-CT analysis of new bone formation in TES. (**G**) Immunofluorescence (IF) imaging of CD31^hi^EMCN^hi^ type-H vessels in tooth extraction socket (TES). Scale bars: 100 μm. (**H**) Panels show the quantitative data of M1-like or M2-like macrophage numbers in the TES (*n* = 5). Results are presented as the mean ± SD.


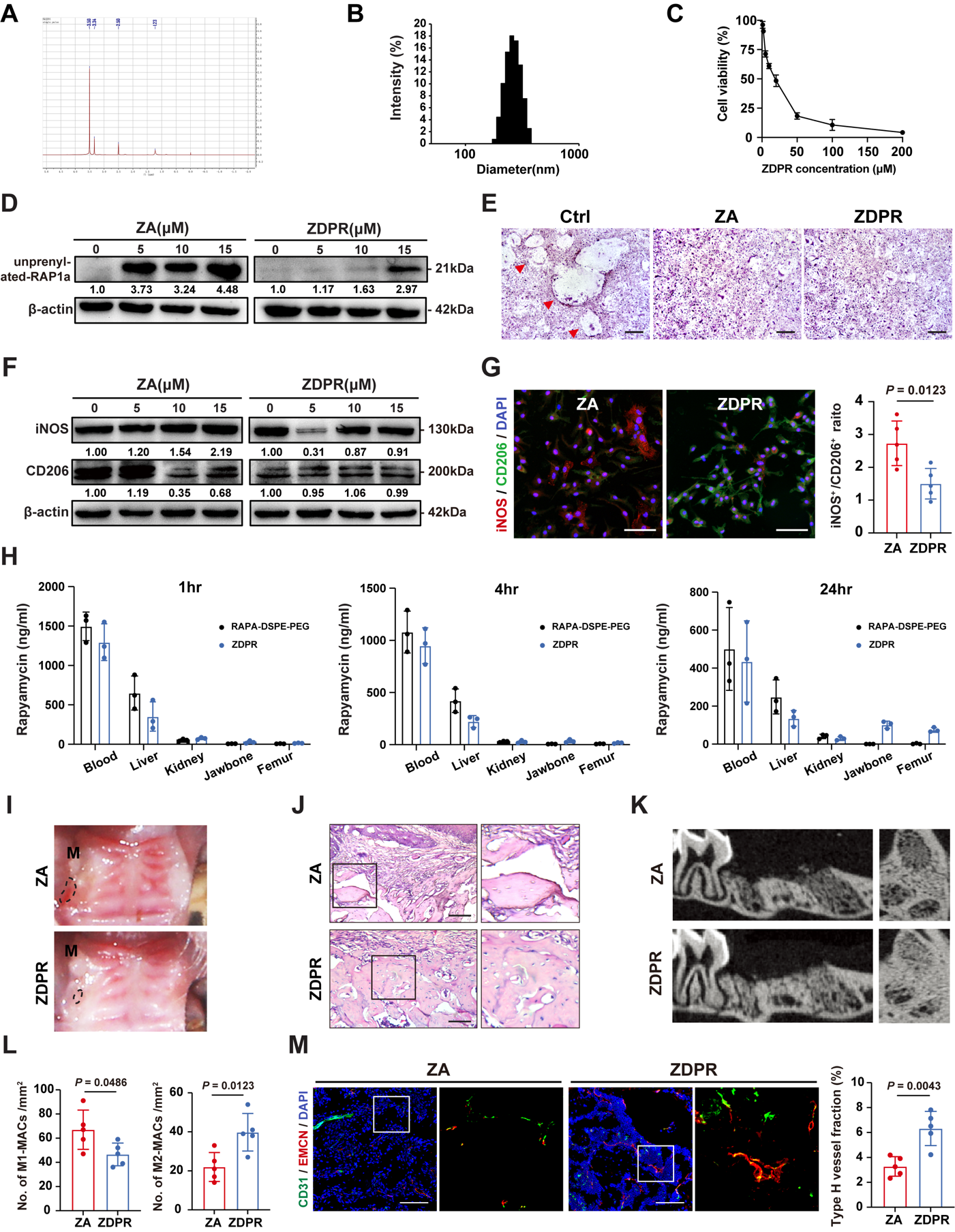


**Fig S6. Properties and treatment effect of ZDPR nanoparticles.** (**A**) NMR of ZA-DSPE_2000_-PEG. (**B**) Measurement of the average diameter of ZDPR nanoparticles. (**C**) CCK-8 assay evaluating the IC_50_ of ZDPR on BMDMs. (**D**) Western blot revealing expression of unprenylated-RAP1a in BMDMs with ZA or ZDPR interventions for 24 hours. (**E**) Representative TRAP staining of osteoclasts differentiation with ZA or ZDPR interventions. (**F**) Western blot revealing iNOS, and CD206 expression in BMDMs with ZA or ZDPR interventions for 24 hours. (**G**) Immunofluorescence (IF) staining detecting the ratio of iNOS positive cells and CD206 positive cells after treatment with ZA or ZDPR. The right panel shows the quantitative measurements of iNOS^+^/ CD206^+^ cell ratio (*n* = 5). Scale bars: 100 μm. (**H**) Biodistribution of rapamycin in whole blood, liver, kidney, jawbone, and femur at 1, 4, 24 hours after DSPE-PEG-RAPA or ZDPR nanoparticles injection. (**I**) Intra-oral photographs depicting wound healing of TES and soft tissue. Dashed lines mark the range of wound adjacent to the second molar (M). (**J**) Representative H&E-stained images of TES. Scale bars: 100 μm. (**K**) Micro-CT analysis of new bone formation in TES. (**L**) Panels show the quantitative data of M1-like or M2-like macrophage numbers in the TES (*n* = 5). (**M**) IF imaging of CD31^hi^EMCN^hi^ type-H vessels in tooth extraction socket (TES). Scale bars: 100 μm. Densitometric quantifications are indicated beneath the representative blots. Results are presented as the mean ± SD.


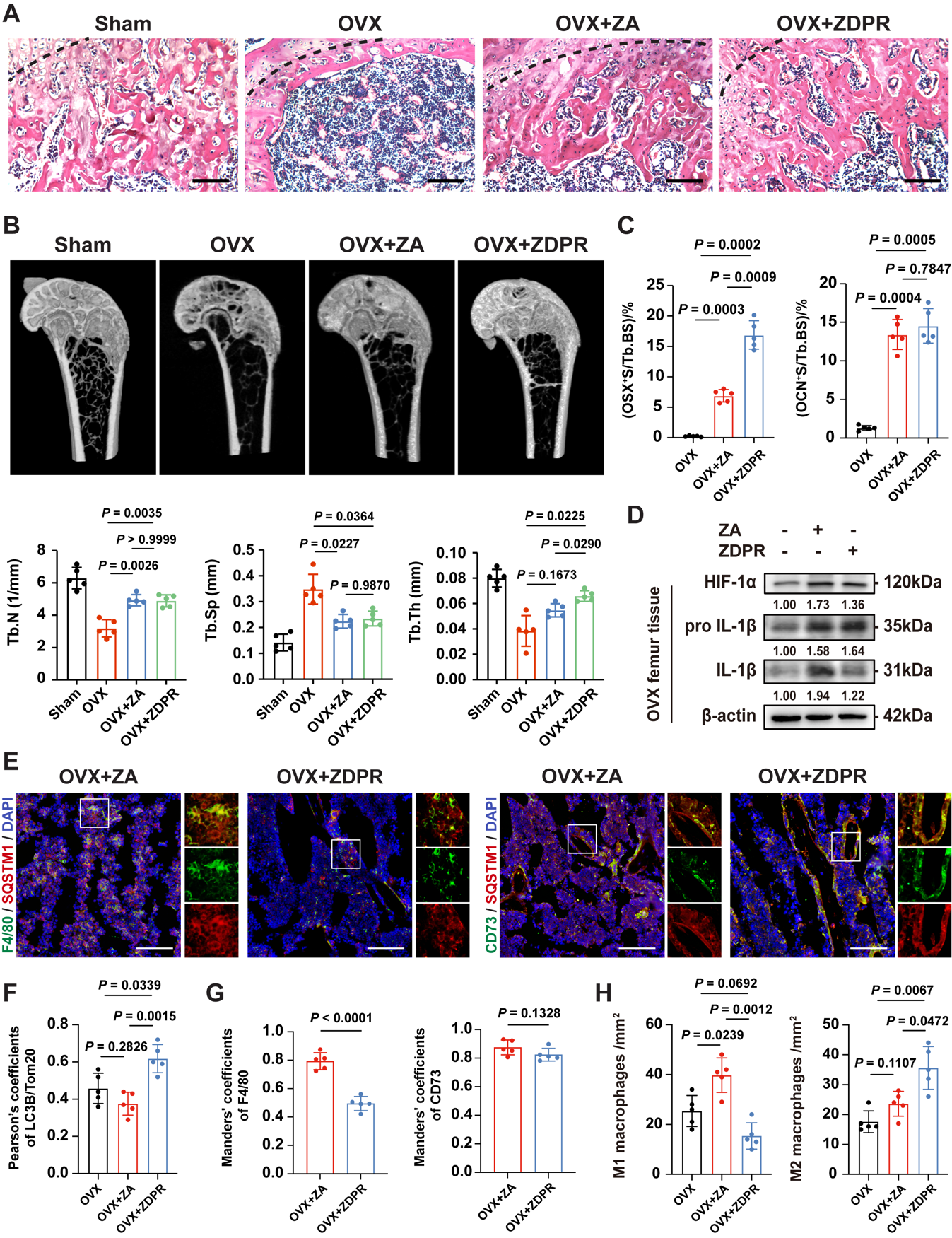
**Figure S7. ZDPR protects against osteoporosis.** (**A**) Representative H&E-stained images of femur 12 weeks after Sham, OVX operation, OVX and ZA injection, or OVX and ZDPR injection. Scale bars: 100 μm. (**B**) Micro-CT analysis of trabecular bone morphology in femur. Panels below show the quantitative measurements of trabecular thickness (Tb.Th), trabecular number (Tb.N), and trabecular separation (Tb.Sp) (*n* = 5). (**C**) Quantification of OSX^+^ or OCN^+^ surface expressed as a percentage of the Tb.BS. (**D**) Westernblot analysis of tissue protein expression in femurs in ZA or ZDPR groups. (**E**) Immunofluorescence staining detecting SQSTM1 expression in F4/80^+^ macrophages or in CD73^+^ mesenchymal stem cells. Scale bars: 100 μm. Panels show quantitative measurements of Pearson’s coefficients for LC3B/Tom20 (**F**, *n* = 5), Mander’s coefficients for F4/80 (**G**, *n* = 5) or CD73 to SQSTM1 (**G**, *n* = 5), and M1-like or M2-like macrophage numbers (**H**, *n* = 5). Densitometric quantifications are indicated beneath the representative blots. Results are presented as the mean ± SD.
